# All Electrochemical Synthesis of Performic Acid Starting from CO_2_, O_2_, and H_2_O

**DOI:** 10.1002/cssc.202500180

**Published:** 2025-04-23

**Authors:** Ida Dinges, Markus Pyschik, Julian Schütz, Selina Schneider, Elias Klemm, Siegfried R. Waldvogel, Markus Stöckl

**Affiliations:** ^1^ Chemical Technology DECHEMA Research Institute Theodor‐Heuss‐Allee 25 60486 Frankfurt am Main Germany; ^2^ Department for Electrosynthesis Max Planck Institute for Chemical Energy Conversion Stiftstraße 34‐36 45470 Mülheim an der Ruhr Germany; ^3^ Institute of Technical Chemistry University of Stuttgart Pfaffenwaldring 55 70569 Stuttgart Germany; ^4^ Institute of Biological and Chemical Systems – Functional Molecular Systems (IBCS‐FMS) Karlsruhe Institute of Technology Kaiserstraße 12 76131 Karlsruhe Germany

**Keywords:** electrochemistry, formic acid, gas diffusion electrode, hydrogen peroxide, peroxides

## Abstract

Driven by anthropogenic climate change, innovative approaches to defossilize the chemical industry are required. Herein, the first all‐electrochemical feasibility study for the complete electrosynthesis of the strong oxidizer and effective disinfectant performic acid is presented. Its synthesis is achieved solely from CO_2_, O_2_, and H_2_O in a two‐step process. Initially, CO_2_ is electrochemically reduced to formate employing Bi_2_O_3_‐based gas diffusion electrodes in a phosphate‐buffered electrolyte. Thereby, high formate concentration (500.7 ± 0.6 mmol L^−1^) and high Faradaic efficiency (86.3 ± 0.3%) are achieved at technically relevant current density (150 mA cm^−2^). Subsequently, the formate acts as (storable) feed electrolyte for the second electrolysis step. Employing carbon‐based gas diffusion electrodes, O_2_ is reduced to H_2_O_2_ and performic acid is directly formed in situ. As before, high H_2_O_2_ concentration (1.27 ± 0.06 mol L^−1^) and high Faradaic efficiency (85.3 ± 5.4%) are achieved. Furthermore, performic acid concentration suitable for disinfection is obtained (82 ± 11 mmol L^−1^). In summary, this innovative feasibility study highlights the potential of combining electrochemical CO_2_ reduction with H_2_O_2_ electrosynthesis, which could provide sustainable access to performic acid in the future.

## Graphical abstract

The first all‐electrochemical synthesis of performic acid starting from CO_2_, O_2_, and H_2_O has been achieved in a two‐step process using gas diffusion electrodes. Therefore, the electrochemical CO_2_ reduction reaction (*eCO*_*2*_ *RR*) to formate was coupled to the electrochemical O_2_ reduction reaction (*eCO*_*2*_ *RR*) to H_2_O_2_ without downstream processing.

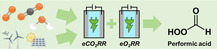

## 1 Introduction

The pressing need for a transition to renewable energy sources and sustainable feedstocks has led to increased research into alternative chemical production processes. Electrochemical processes, in particular, are emerging as a promising route for achieving this goal, enabling the direct conversion of renewable electricity into value‐added chemical products.^[^ ^1^, ^2^, ^3^, ^4^ ^]^ Such approaches offer not only a reduction in process emissions, but also the potential to integrate decentralized, onsite manufacturing that can complement renewable energy and systems.

One of the most compelling areas of electrochemical synthesis is the reduction of carbon dioxide (CO_2_) and its implementation as a renewable feedstock in the chemical industry. Among the various products generated by the electrochemical CO_2_ reduction reaction (*eCO*_*2*_ *RR*), formic acid and formate represent attractive intermediates due to their good storage and transport properties, as well as their versatile applications.^[^ ^5^, ^6^, ^7^, ^8^, ^9^ ^]^ Thereby, the aqueous *eCO*_*2*_ *RR* can be performed efficiently using gas diffusion electrodes (GDEs), allowing high current densities up to 1.8 A cm^−2^,^[^ ^10^ ^]^ Faradaic efficiencies (FE) exceeding 90%,^[^ ^11^, ^12^, ^13^, ^14^ ^]^ and operational times of 1000 h.^[^ ^15^ ^]^

Beyond its immediate use, formic acid/formate can serve as versatile platform chemical for synthesizing higher‐value products combining subsequent chemical and biological processes. Exemplarily, the use as a feedstock in microbial synthesis offers the option to produce more complex and attractive products,^[^ ^16^, ^17^, ^18^, ^19^ ^]^ which has been demonstrated exemplarily for the synthesis of the biopolymer polyhydroxybutyrate (PHB) without the need for further downstream processing between *eCO*_*2*_ *RR* and the biosynthesis.^[^ ^8^, ^9^ ^]^ Another pathway for formic acid/formate is its utilization for the synthesis of the strong oxidizer and disinfectant performic acid (PFA),^[^ ^20^, ^21^, ^22^, ^23^ ^]^ which is obtained in the equilibrium reaction of formic acid and hydrogen peroxide. PFA is an attractive disinfectant for several sectors such as the food industry, healthcare, and wastewater treatment, as it is already very effective at low concentrations (<20 mg L^−1^ ≙ 0.32 mmol L^−1^).^[^ ^24^, ^25^, ^26^, ^27^ ^]^ It is also environmentally friendly, especially compared to halogen‐based disinfectants. Decomposition leads to CO_2_, O_2_ and H_2_O whereas the formation of harmful disinfection byproducts is largely unlikely.^[^ ^27^, ^28^, ^29^, ^30^, ^31^ ^]^ The downside of its high reactivity is that it is not stable and safe enough for storage and needs to be produced onsite shortly before use from formic acid and H_2_O_2_.^[^ ^26^, ^31^, ^32^, ^33^, ^34^, ^35^ ^]^

The traditional industrial anthraquinone process for H_2_O_2_ production is energy intensive, generates organic waste, and is associated with significant CO_2_ emissions.^[^ ^36^ ^]^ Facing these challenges, H_2_O_2_ syntheses via the two‐electron oxygen reduction reaction (*eO*_*2*_ *RR*) using GDEs has emerged as an alternative approach to the anthraquinone process. Thereby, FE above 90%,^[^ ^37^, ^38^, ^39^ ^]^ current densities up to 0.5 A cm^−2^,^[^ ^39^ ^]^ and operational times in range of 200–1000 h^[^ ^40^, ^41^, ^42^ ^]^ have been already reported.

If H_2_O_2_ is synthesized electrochemically in the presence of carboxylic acids such as formic acid, PFA is generated in situ. Recently, Schneider and Stöckl^[^ ^23^ ^]^ published a comprehensive approach to the indirect and on‐demand electrosynthesis of various peroxy acids via in situ generated H_2_O_2_ on GDEs, including PFA. After 24 h of electrosynthesis in a buffered system with 1 wt% H_3_PO_4_, 1.47 mol L^−1^ H_2_O_2_, and 0.24 mol L^−1^ PFA have been obtained with FEs of 37.5% and 6.1% (combined FE = 43.6%), respectively.

**Figure 1:**
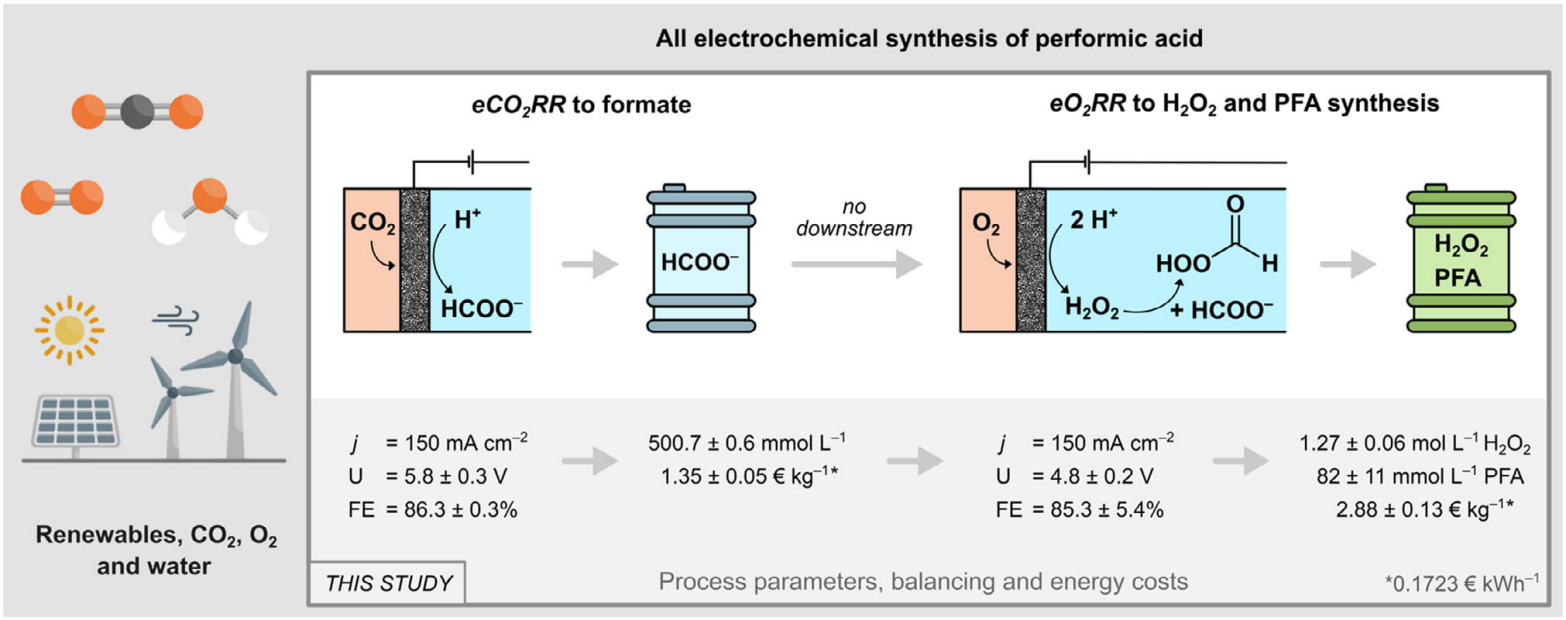
Schematic illustration and key process data of the overall process combination of the electrochemical CO_2_ reduction reaction (*eCO*_*2*_ *RR*) to formate with the electrochemical O_2_ reduction reaction (*eO*_*2*_ *RR*) to H_2_O_2_ to synthesize PFA using renewable energy sources. Resulting costs for formate and H_2_O_2_/PFA were based on a recent energy price.^[^ ^47^ ^]^ Abbreviations: *j* = current density, *U* = average cell voltage, FE = Faradaic efficiency.

With this study, the first innovative all‐electrochemical feasibility study for the complete electrosynthesis of PFA from CO_2_, O_2_, and H_2_O through a two‐step process utilizing GDEs is presented (cf. **Figure** **1**). In the first step, CO_2_ is electrochemically reduced to formate, which then acts as (storable) feed electrolyte in the second step, where O_2_ is reduced to H_2_O_2_ and PFA is formed in situ. The authors thereby continue the findings of their previous study, where it was shown that phosphate ions/phosphoric acid and a buffered electrolyte are beneficial for PFA synthesis,^[^ ^23^ ^]^ leading to the initial *eCO*_*2*_ *RR* in a phosphate‐buffered electrolyte solution.

This approach not only highlights the potential of combining CO_2_ reduction with H_2_O_2_ synthesis, but demonstrates the advantages of electrochemical processes like decentralized and on‐demand production using the example of PFA, a highly effective oxidant and disinfectant that is not stable and safe enough for storage.

## 2 Results and Discussion

### 2.1 GDE Fabrication and Characterization for *eCO*_*2*_ *RR* and *eO*_*2*_ *RR*

GDEs for *eCO*_*2*_ *RR* were fabricated by heat pressing a catalyst mixture onto Ni foam as support material and current collector according to the oxygen‐depolarized cathode (ODC) technology developed by Covestro.^[^ ^43^ ^]^ The catalyst mixture consisted of Bi_2_O_3_ nanopowder (≈200 € kg^−1^) as electrocatalyst and polytetrafluoroethylene (PTFE, ≈50 € kg^−1^) powder as a hydrophobic binder. Taking contemporary costs into account, the GDE's material cost was estimated at 474 € m^−2^, of which Ni foam (≈338 € m^−2^) accounts for 71.3%. In total, three GDEs (*n* = 3) were fabricated for formate feed generation via *eCO*_*2*_ *RR*. The reproducibility of the predominantly manual fabrication method was sufficient, resulting in GDEs with catalyst loading *b* (Bi_2_O_3_, wt%) = 65.7 ± 0.7 mg cm^−2^ and thickness *d* = 523 ± 11 μm (*n* = 3). Furthermore, no influence of minor fabrication variations on GDE performance was observed.

GDEs for *eO*_*2*_ *RR* were fabricated in a similar manner as described above, their composition and part of the pressing protocol (excluding sintering at 340 °C) was based on Kopljar et al.^[^ ^44^ ^]^ The catalyst mixture consisted of acetylene black powder (≈320 € kg^−1^) as carbon‐based electrocatalyst and PTFE powder as binder. The mixture was pressed onto stainless steel mesh (≈10 € m^−2^) using a cylindrical mask. Afterward, GDEs were treated in a heat press to improve their mechanical stability. In total, eight GDEs (*n* = 8) were fabricated with a catalyst loading of *b* = 26.1 ± 0.5 mg cm^−2^ and a thickness of *d* ≈ 600 ± 10 μm. Based on recent prices, the GDE's material cost was estimated at 62 € m^−2^.

**Figure 2:**
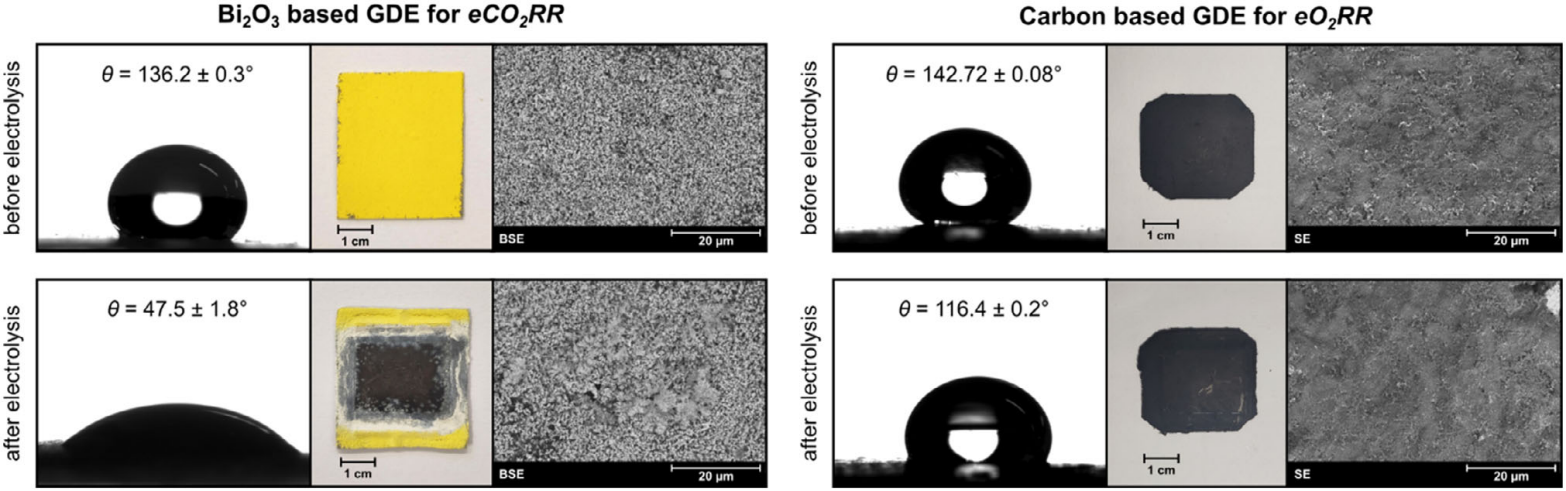
Fabricated GDE for *eCO*_*2*_ *RR* (left) and *eO*_*2*_ *RR* (right) and their characterization before and after electrolysis using SEM imaging (BSE = back scattering electrons, SE = secondary electrons) and contact angle measurements.

Both fabricated GDE types were characterized before and after electrolysis by contact angle measurement, density measurement, and scanning electron microscopy (SEM). Exemplary results are summarized in **Figure** **2**.

The pristine Bi_2_O_3_ GDE was bright yellow in color, had a contact angle of 136.2 ± 0.3° (*n* = 2) and a density of 7.060 ± 0.002 g cm^−3^ (*n* = 3), and showed evenly distributed, mostly round Bi_2_O_3_ particles in the SEM (back‐scattering electrons) image. After electrolysis, the GDE showed a black discoloration of the area exposed in the flow cell during electrolysis. This was attributed to the reduction of Bi_2_O_3_ to elemental Bi at the beginning of the electrolysis, which was supported by X‐ray diffraction results (cf. Supporting Information). Accordingly, the discolored GDE area had a lower density of 6.631 ± 0.004 g cm^−3^ (*n* = 3). The discoloration was further examined using SEM, whereby new dendritic structures were observed. Alongside or in addition to Bi_2_O_3_ reduction, cathodic corrosion^[^ ^45^ ^]^ could have contributed to the change in the GDE's surface structure. Therefore, GDE stability was investigated and will be discussed later regarding feed characterization. Apart from optical changes, the GDE's contact angle decreased significantly to 47.5 ± 1.8° (*n* = 2) after electrolysis. It therefore became relatively hydrophilic during electrolysis, which was expected due to electrowetting.

The carbon GDE for *eO*_*2*_ *RR* did not show significant optical differences or changes in the GDE surface via SEM either before or after electrolysis. However, the contact angle changed from 142.72 ± 0.08° (*n* = 2) to 116.4 ± 0.2° (*n* = 2) after electrolysis. Consequently, this GDE type was also wetted during electrolysis but remained relatively hydrophobic compared to Bi_2_O_3_ GDEs. Moreover, the initial GDE density of 3.094 ± 0.004 g cm^−3^ (*n* = 3) increased to 3.270 ± 0.001 g cm^−3^ (*n* = 3). This density increase might have been caused by salt residues within the GDE, although no significant GDE swelling was observed.

### 2.2 *eCO*_*2*_ *RR* to Formate

**Figure 3:**
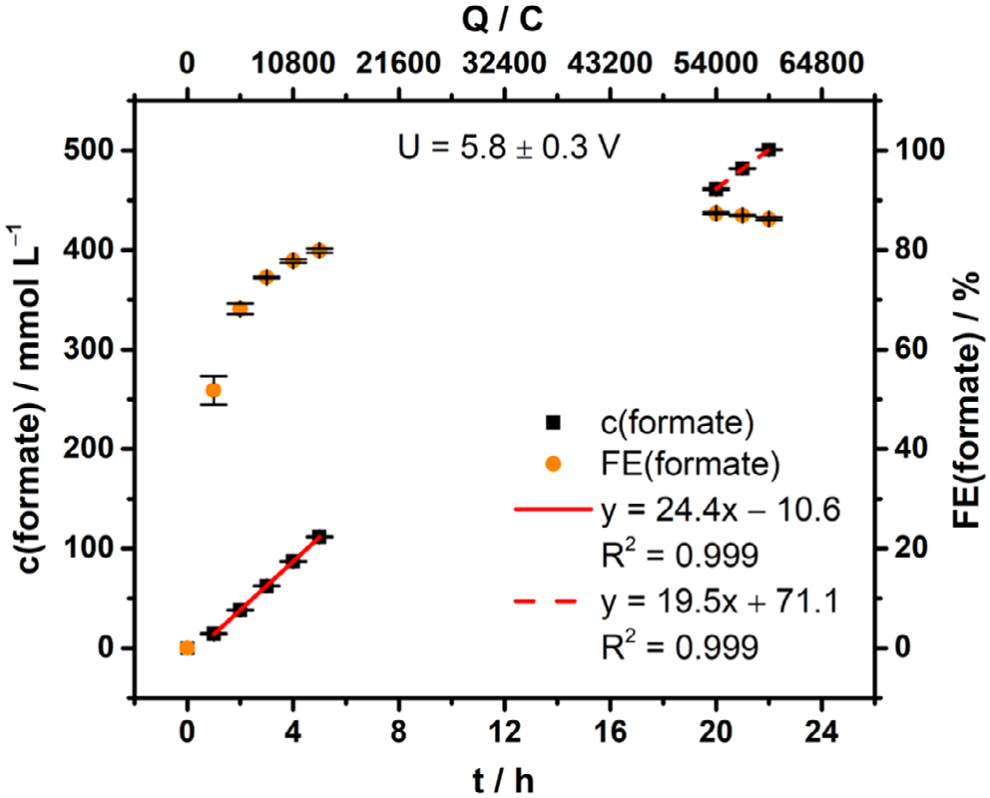
Concentration and FE course of formate for formate electrosynthesis (*n* = 3). The formate concentration course was fitted linearly in two intervals: *t* = 1–5 h (red, solid line) and *t* = 20–22 h (red, dashed line). Electrolysis parameters: constant current density *j* = 150 mA cm^−2^, runtime = 22 h (≙59,400 C), electrolyte = 0.2 mol L^−1^ KH_2_PO_4_/K_2_HPO_4_, initial *V* (catholyte, anolyte) = 500 mL each, cathode (GDE) = 87.5 wt% Bi_2_O_3_, 12.5 wt% PTFE on Ni‐foam, reference electrode = reversible hydrogen electrode (RHE), anode = mixed Ir‐oxide on a Ti‐grid (Platinode EP, Type 177, Umicore).

To generate the formate feed for subsequent H_2_O_2_ electrosynthesis and PFA generation, *eCO*_*2*_ *RR* was carried out with Bi_2_O_3_ GDEs in a gas‐fed flow reactor (divided cell, cf. Experimental Section) using 0.2 mol L^−1^ KH_2_PO_4_/K_2_HPO_4_ (equimolar, pH ≈ 6.67) as both anolyte and catholyte. Phosphate buffer was chosen as a supporting electrolyte based on the results from Schneider and Stöckl^[^ ^23^ ^]^ for peroxy acid synthesis using electrochemically generated H_2_O_2_. Furthermore, promising results were obtained using this electrolyte with Sn‐based GDE.^[^ ^9^ ^]^ Generally, the aim was to generate a feed with high formate concentration (>0.5 mol L^−1^) at high FE (>85%). For this purpose, all electrolyses were run 22 h at 150 mA cm^−2^. **Figure** **3** contains the courses of formate concentration and FE in the catholyte.

Formate concentration increased linearly over time, its course was fitted in two intervals to obtain the respective formate production rate. It shows the rate declined about 20% from 24.4 ± 0.2 mmol L^−1^ h^−1^ (interval 1 = 1–5 h, ≙113 ± 1 mg h^−1^ cm^−2^) to 19.5 ± 0.7 mmol L^−1^ h^−1^ (interval 2 = 20–22 h, ≙89.8 ± 3.2 mg h^−1^ cm^−2^) during runtime. This decline could be attributed to presumed formate mass transport limitations within the GDE's pore system, which would increase the influence of the parasitic hydrogen evolution reaction (HER). Additionally, the decline could also be caused by a possible decrease in the GDE's stability during electrolysis (cf. feed characterization). Nonetheless, a final formate concentration of 500.7 ± 0.6 mmol L^−1^ (*n* = 3) was achieved.

Moreover, formate FE shows a nonlinear increase in the first 5 h runtime, especially the FE at 1 h is rather low with 52 ± 3% (*n* = 3). On the one hand, this is likely caused by partial consumption of supplied charge to reduce Bi_2_O_3_ to Bi. On the other hand, the GDE's electrowetting behavior could have contributed to the low initial FE. Nonetheless, to reach an overall FE of 79.9 ± 0.5% (*n* = 3) during the first 5 h, the FE per hour must have been close to 90% after the initial first hour. Consequently, the Bi_2_O_3_ type GDEs fabricated herein have a peak FE performance of close to 90%. Overall, 86.3 ± 0.3% (*n* = 3) was achieved as final formate FE in 22 h runtime.

Regarding energy demand, electrolyses were run with an average cell voltage of 5.8 ± 0.3 V (*n* = 3). This relatively high cell voltage was mainly caused by ohmic losses in the anode chamber, as a nonzero‐gap anode was used for the oxygen evolution reaction (OER) as counter reaction, the GDEs’ average potential was only −1.21 + 0.03 V versus RHE (*n* = 3, cf. Supporting Information). However, the GDEs’ potential is also relatively high, possibly due to bismuth's low electric conductivity (0.936 × 10^6^ S m^−1^ at 298 K).^[^ ^46^ ^]^ In total, electrolyses consumed 96 ± 4 Wh (*n* = 3) of electric energy. Hence, formate electrosynthesis required 7.9 ± 0.3 kWh kg^−1^, which corresponds to 1.35 ± 0.05 € kg^−1^ formate (no downstream processing etc., 0.1723 € kWh^−1^,^[^ ^47^ ^]^ cf. Figure 1). This is higher than market prices for fossil‐based concentrated formic acid such as 0.37 and 0.69 € kg^−1^ (0.40 $ kg^−1^^[^ ^48^ ^]^ and 0.74 $ kg^−1^,^[^ ^49^ ^]^ respectively with 1 € ≙ 1.08 $). Consequently, lowering cell voltage and thereby energy demand of *eCO*_*2*_ *RR* are essential to compete with current fossil‐based market prices. As discussed above, the integration of a zero‐gap anode could lower cell voltage significantly.

### 2.3 *eO*_*2*_ *RR* to H_2_O_2_ and PFA Synthesis

In the next step, the generated formate feed (pH ≈ 4.12, formic acid pK_a_ = 3.75 at 25 °C)^[^ ^50^ ^]^ was used as catholyte for H_2_O_2_ electrosynthesis and PFA generation. It was carried out with the same flow cell setup as above, but employing carbon‐based GDEs.

**Figure 4:**
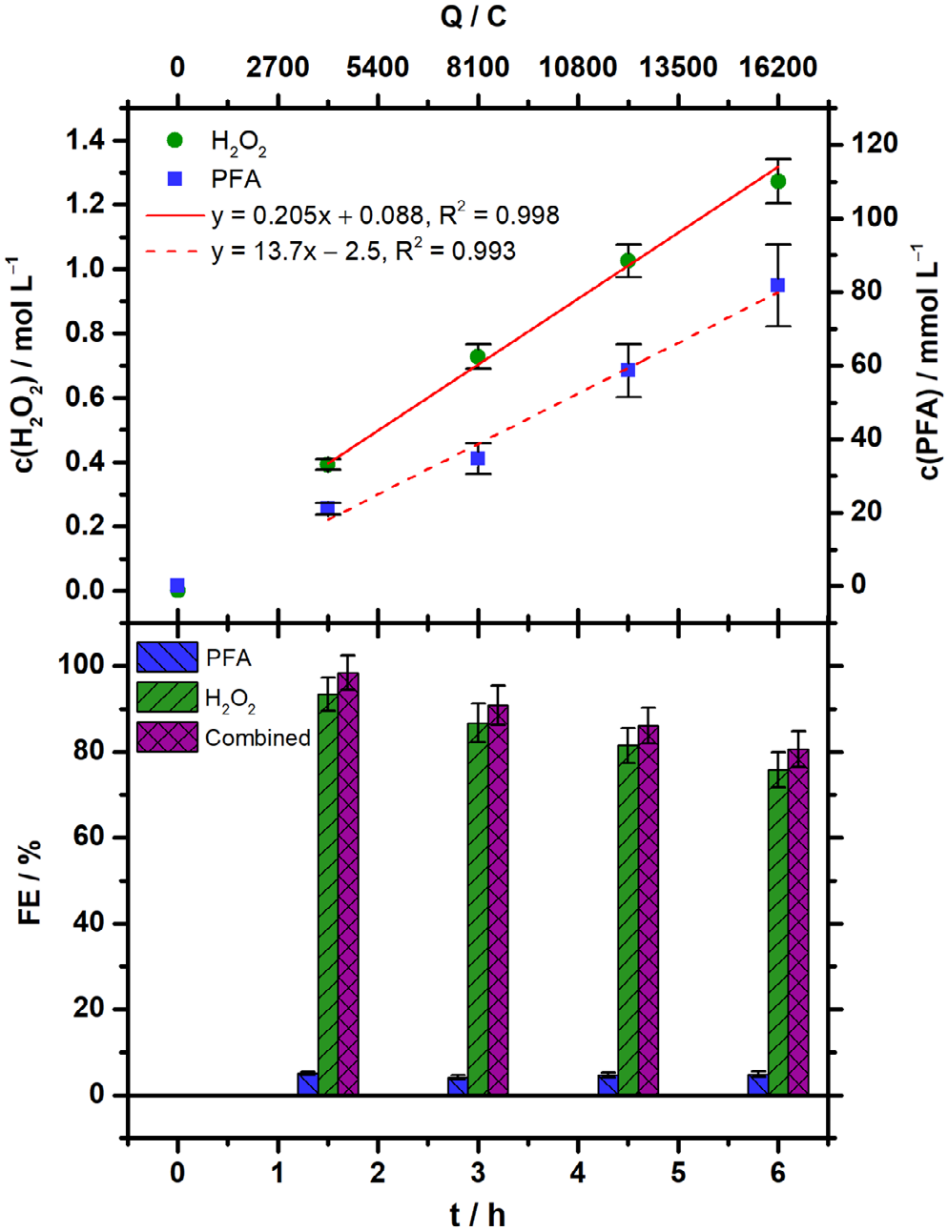
Concentration and FE course of H_2_O_2_ and PFA for H_2_O_2_ electrosynthesis and PFA generation (*n* = 4). Electrolysis parameters: constant current density *j* = 150 mA cm^−2^, runtime = 6 h (≙16,200 C), catholyte = formate containing catholyte originating from *eCO*_*2*_ *RR* to formate (50 mL), anolyte = 0.5 mol L^−1^ HClO_4_ (50 mL), cathode (GDE) = 65.5 wt% acetylene black, 34.5 wt% PTFE on stainless steel mesh, reference electrode = reversible hydrogen electrode (RHE), anode = mixed Ir‐oxide on a Ti‐grid (Platinode EP, Type 177, Umicore).

All electrolyses were run 6 h at 150 mA cm^−2^, catholyte samples were taken every 1.5 h to determine H_2_O_2_ and PFA (pK_a_ = 7.3 at 25 °C)^[^ ^50^ ^]^ concentrations by two‐step titration^[^ ^51^ ^]^ (H_2_O_2_ was first quantified by cerimetry and then PFA by iodometry, *n* = 3 for each sample). Direct FE for H_2_O_2_ and indirect FE for PFA were calculated based on their determined amounts and under the assumption that H_2_O_2_ and formate react equimolar with each other to form PFA (cf. Equation (2), Experimental Section). A rapid adjustment of the reaction's equilibrium was assumed based on Schneider and Stöckl^[^ ^23^ ^]^ results. The combined FE therefore corresponds to the actual FE of H_2_O_2_ electrosynthesis. **Figure** **4** summarizes the concentration and corresponding FE courses obtained during electrolysis (*n* = 4).

H_2_O_2_ concentration increased linearly over time up to 1.27 ± 0.06 mol L^−1^ (*n* = 4) in 6 h. After initial GDE conditioning, a production rate of 20.5 ± 0.9 mmol L^−1^ h^−1^ (≙6.97 ± 0.31 mg h^−1^ cm^−2^) was reached (*t* = 1.5–6 h). This was accompanied by a continuous increase in PFA concentration (13.7 ± 1.2 mmol L^−1^ h^−1^, *t* = 1.5–6 h) to finally 82 ± 11 mmol L^−1^ (*n* = 4). Despite the concentration's increase, H_2_O_2_ FE declined by about 17% from initially 93 ± 4% (*n* = 4) down to 76 ± 5% (*n* = 4) during runtime (50 mL catholyte volume assumed). This also applies to the combined FE, so the actual H_2_O_2_ FE, as the PFA's indirect FE remains nearly constant at 4–5% due to its reaction equilibrium. However, the final H_2_O_2_ FE had to be corrected to account for an observed increase in catholyte volume after electrolysis. Thereby, a combined H_2_O_2_ FE of 85.3 ± 5.4% (*n* = 4) was achieved in 6 h runtime. The FE balance is most likely closed by parasitic HER, but four‐electron oxygen reduction to H_2_O and/or H_2_O_2_ decomposition during electrolysis cannot be fully excluded. However, significant decomposition of H_2_O_2_/PFA after electrolysis was not indicated (e.g., gas evolution/titration results, cf. Supporting Information).

Electrolyses were run with an average cell voltage of 4.8 ± 0.2 V (*n* = 4) consuming 21.5 ± 0.6 Wh (*n* = 4) of electric energy. As before, this is a relatively high cell voltage caused by the ohmic losses due to OER at the nonzero‐gap anode, the GDEs’ potential was only −1.4 + 0.5 V versus RHE (*n* = 4, cf. Supporting Information). Consequently, H_2_O_2_ required 8.9 ± 0.6 kWh kg^−1^ (*n* = 4) corresponding to 1.53 ± 0.11 € kg^−1^ as electric energy cost (no downstream processing etc., 0.1723 € kWh^−1^,^[^ ^47^ ^]^ cf. Figure 1). In comparison, fossil‐based market prices for concentrated H_2_O_2_ are in the range of 0.64–1.1 € kg^−1^ (700–1200 $ t^−1^, with 1 ≙ 1.08 $).^[^ ^52^ ^]^ For the target byproduct PFA, a yield of 16.3 ± 2.2% (*n* = 4) was obtained. To achieve higher PFA concentrations/yields, higher concentrations of H_2_O_2_ and formate are necessary. This is also supported by Greenspan's^[^ ^53^ ^]^ results on PFA formation. Taking presumed formate and H_2_O_2_ energy costs into account, the H_2_O_2_/PFA product solution cost added up to 2.88 ± 0.13 € kg^−1^ (*n* = 4). As PFA is usually generated onsite of its application, no commercial price was available as reference.

For comparison, H_2_O_2_ electrosynthesis for PFA generation was also performed with a reference electrolyte (R) based on the formate feed generated via *eCO*_*2*_ *RR*. It was prepared from commercially available compounds and contained 0.2 mol L^−1^ KH_2_PO_4_/K_2_HPO_4_ (equimolar) and 0.5 mol L^−1^ HCOOK/HCOOH (equimolar) in 1 wt% H_3_PO_4_. It was adjusted to pH = 4.13 ± 0.05 with KOH. Using (R) as catholyte, H_2_O_2_ and PFA showed concentration and FE courses similar to the results presented above, whereas overall values were slightly lower. In short, a combined H_2_O_2_ FE of 78 ± 7% (*n* = 4) was obtained in combination with 13.7 ± 0.8% (*n* = 4) PFA yield. Hence, the formate feed can be generated via *eCO*_*2*_ *RR* with slight improvements instead of performance losses for H_2_O_2_ electrosynthesis and PFA generation. Details are provided (Section S3.2.2, Supporting Information).

So far, only a few results have been published on (indirect) electrochemical PFA synthesis. Recently, Sun et al.^[^ ^54^ ^]^ conceptually reported an all‐electrochemical PFA synthesis with parallel *eCO*_*2*_ *RR* and *eO*_*2*_ *RR* using their trifunctional indium‐based catalyst/electrolysis system. Furthermore, Kolyagin et al.^[^ ^20^ ^]^ used 1.5 mol L^−1^ formic acid (+0.2 mol L^−1^ K_2_SO_4_ and 0.05 mol L^−1^ H_2_SO_4_) as electrolyte solution for *eO*_*2*_ *RR* to H_2_O_2_ with a carbon‐based GDE. Thereby, they achieved 1.8 mol L^−1^ H_2_O_2_ (55% FE) and 6.5 mmol L^−1^ PFA in 7 h electrolysis at 100 mA cm^−2^.

Schneider and Stöckl^[^ ^23^ ^]^ recently published a new benchmark for the indirect electrosynthesis of PFA among various peroxy acids via in situ generated H_2_O_2_ on commercial carbon black GDEs, as mentioned in the introduction. Thereby, 0.77 mol L^−1^ H_2_O_2_ (69% FE) and 0.14 mol L^−1^ PFA (13% FE) were achieved in 6 h at 100 mA cm^−2^ with 1 mol L^−1^ HCOOH/HCOOK (+1 wt% H_3_PO_4_) as electrolyte solution. This corresponded to a combined FE of 82% for H_2_O_2_ electrosynthesis. Compared to these previous results, H_2_O_2_ concentration (1.27 ± 0.06 mol L^−1^) and combined FE (85.3 ± 5.4%) were improved at increased current density (150 mA cm^−2^) herein. In contrast, PFA concentration (82 ± 11 mmol L^−1^) was lower, which is most probably attributed to the lower initial formate concentration of the formate feed used as electrolyte solution. Nonetheless, 82 ± 11 mmol L^−1^ PFA represents the first benchmark for an all‐electrochemical approach to PFA synthesis using CO_2_, O_2_, and H_2_O to the best of the authors’ knowledge. Even though there is space for improvement in terms of electrolysis and reaction/equilibrium adjustment to reach higher PFA concentrations, the obtained PFA/H_2_O_2_ mixture could most likely already be applied for disinfection. On the one hand, far lower PFA concentrations (<20 mg L^−1^ ≙ 0.32 mmol L^−1^)^[^ ^24^, ^26^, ^27^ ^]^ have been reported as sufficient for disinfection. On the other hand, a mixture of H_2_O_2_, formic acid, and PFA could be advantageous, as synergistic effects have been reported for comparable mixtures.^[^ ^55^ ^]^

### 2.4 Feed Characterization/GDE Evaluation

After electrolysis, both formate feed and H_2_O_2_/PFA product solution were further examined for characterization and to evaluate GDE stability. On the one hand, K^+^ and PO_4_^3−^ concentrations were determined by ion chromatography (IC) to examine possible differences compared to their initial concentrations. These results were also used to prepare reference electrolyte (R) as formate feed substitute for H_2_O_2_ electrosynthesis and PFA generation. On the other hand, all solutions were screened via inductively coupled plasma atomic emission spectroscopy (ICP‐OES) for metal ions originating from the GDEs as well as other impurities that could decompose H_2_O_2_/PFA. Detected target elements were Bi (electrocatalyst *eCO*_*2*_ *RR*), Ni (support material *eCO*_*2*_ *RR*/*eO*_*2*_ *RR*), Cr and Fe (support material *eO*_*2*_ *RR*, respectively). Hence, their concentrations were quantified via calibration to investigate cathodic corrosion^[^ ^45^ ^]^ and thus GDE stability. The characterization results are summarized in **Table** **1**.

**Table 1:** Catholyte/feed composition before and after formate as well as H_2_O_2_ electrosynthesis for PFA generation. K^+^ and PO_4_^3−^ concentrations were determined via IC and Bi^3+^, Cr^3+^, Fe^3+^, and Ni^2+^ concentrations were quantified via ICP‐OES.

	*c*(K^+^) [mmol L^−1^]	*c*(PO_4_^3−^) [mmol L^−1^]	*c*(Bi^3+^) [μmol L^−1^]	*c*(Cr^3+^) [μmol L^−1^]	*c*(Fe^3+^) [μmol L^−1^]	*c*(Ni^2+^) [μmol L^−1^]
0.2 mol L^−1^ KH_2_PO_4_/K_2_HPO_4_ (*n* = 1)	286.2	197.5	0	0.270 ± 0.013	2.71 ± 0.05	0.13 ± 0.04
Formate feed (*n* = 3)	542 ± 6	183.4 ± 0.4	0.6 ± 0.4	0.224 ± 0.004	1.67 ± 0.15	0.41 ± 0.17
H_2_O_2_/PFA solution (*n* = 4)	423 ± 28	147 ± 10	0.18 ± 0.06	0.23 ± 0.04	1.83 ± 0.14	0.55 ± 0.19

IC analysis revealed the formate feed's PO_4_^3−^ concentration was slightly lower (7%) than the initial phosphate buffer, which is due to an increase in volume by osmosis during electrolysis. Moreover, PO_4_^3−^ concentration decreases further by about 20% during *eO*_*2*_ *RR*. This decrease cannot be explained solely by a volume increase or by losses due to a concentration gradient to the anolyte (HClO_4_), as the anolyte's phosphate concentrations remained below 0.8 mmol L^−1^ (cf. Section S1.12, Supporting Information).

Besides, K^+^ concentration was almost doubled after *eCO*_*2*_ *RR*. Nearly all K^+^ ions are found in the formate feed due to migration, their molar amount in the electrolyte system was constant. In contrast, K^+^ concentration was about 22% lower in the H_2_O_2_/PFA product solution. Some K^+^ was found in the anolyte (12.6 ± 1.8 mmol L^−1^, *n* = 3) but its molar amount was not constant.

Although no GDE swelling was observed, it was presumed both K^+^ and PO_4_^3−^ discrepancies were caused by salt precipitation within the carbon‐based GDE. This hypothesis is supported by the GDEs’ increased density after *eO*_*2*_ *RR* as discussed previously.

For ICP‐OES analysis, the phosphate buffer was examined before characterization of formate feed and product solution. It already contained traces of the identified target elements Cr, Fe, and Ni, which originate from the compounds used for electrolyte preparation. Besides, ICP‐OES analysis found only traces of dissolved Bi in the formate feed after *eCO*_*2*_ *RR*. Its concentration was even lower after *eO*_*2*_ *RR* due to dilution, as expected for carbon‐based GDEs. In general, these traces were above detection but below quantification limit, which resulted in relatively high standard deviations (30–40%). Furthermore, quantification was challenging due to strong matrix effects, as it was observed that Bi^3+^ partly precipitated as BiPO_4_ in standards containing matrix (cf. Section S1.11, Supporting Information). Consequently, most BiPO_4_ could have already precipitated during electrolysis, although no precipitates were observed. Therefore, the extent of Bi's cathodic corrosion cannot be fully assessed but is most likely overall very low, as other studies on Bi as electrocatalyst also suggest.^[^ ^56^ ^]^ Additionally, the previously discussed possible loss of GDE stability can neither be confirmed nor ruled out as explanation for performance decline during electrolysis. However, as the formate feed's Bi^3+^ concentration was at least below 1 μmol L^−1^, a significant influence on H_2_O_2_/PFA generation seemed unlikely. Moreover, the feed's Cr^3+^ and Fe^3+^ concentrations were lower compared to their initial ones due to dilution. In contrast, Ni^2+^ concentration was increased by 32% to 0.41 ± 0.17 μmol L^−1^ (*n* = 3). This shows the Bi_2_O_3_ GDE's support material Ni foam was in contact with catholyte solution and was slightly affected by cathodic corrosion.

Further analysis of the H_2_O_2_/PFA solution after *eO*_*2*_ *RR* revealed Cr^3+^ concentration remained relatively constant, while Fe^3+^ and Ni^2+^ concentrations increased slightly. As the feed is diluted during electrolysis (≈5%), molar amounts of all three elements were increased by cathodic corrosion of the stainless steel mesh (1.4301) serving as support material. Nonetheless, all concentrations were rather low and did not seem to have compromised H_2_O_2_/PFA generation, especially in the presence of phosphate ions acting as possible chelating agents.^[^ ^57^ ^]^

However, the future aim should be to minimize overall metal ion impurities to avoid potential efficiency losses, as these could potentially catalyze H_2_O_2_/PFA decomposition.^[^ ^31^, ^58^, ^59^, ^60^ ^]^

Furthermore, Schneider and Stöckl^[^ ^23^ ^]^ hypothesized that leached Ni may have affected their H_2_O_2_ electrosynthesis for PFA generation. Although only a total of 0.55 ± 0.19 μmol L^−1^ Ni was leached from the self‐fabricated GDEs presented, future GDEs should ideally be realized without metallic support material (without compromising performance and stability).

## 3 Conclusion

In this study, the first all‐electrochemical PFA synthesis has been achieved by coupling *eCO*_*2*_ *RR* to formate with *eO*_*2*_ *RR* to H_2_O_2_ without any intermediate downstream processing.

The formate feed was generated as catholyte by electrochemical CO_2_ reduction using Bi_2_O_3_ GDEs (474 € m^−2^), which were self‐fabricated by a fast, facile, and reproducible fabrication method. The GDEs were operated at 150 mA cm^−2^ for 22 h to generate a feed containing 500.7 ± 0.6 mmol L^−1^ (*n* = 3) formate with a pH of 4.12. Thereby, an overall formate FE of 86.3 ± 0.3% (*n* = 3) was achieved, while a peak FE up to 90% was reached. Furthermore, no significant cathodic corrosion was observed for the GDEs, Bi^3+^ concentration was at least below 1 μmol L^−1^, and Ni^2+^ concentration was below 0.6 μmol L^−1^.

The subsequent *eO*_*2*_ *RR* to H_2_O_2_ was carried out with the formate feed as catholyte at self‐fabricated, inexpensive carbon GDEs (62 € m^−2^). They were operated at 150 mA cm^−2^ for 6 h without significant cathodic corrosion, whereby 1.27 ± 0.06 mol L^−1^ (*n* = 4) H_2_O_2_ and 82 ± 11 mmol L^−1^ (*n* = 4) PFA were reached. This corresponded to an overall H_2_O_2_ FE of 85.3 ± 5.4% (*n* = 4) and a PFA yield of 16.3 ± 2.2% (*n* = 4). The achieved PFA concentration represents the first benchmark for an all‐electrochemical PFA synthesis to the best of the authors’ knowledge. Furthermore, it already exceeds concentrations suitable for disinfection (<20 mg L^−1^ ≙ 0.32 mmol L^−1^).^[^ ^24^, ^26^, ^27^ ^]^

Finally, electric energy costs of the overall process were assessed for formate (1.35 ± 0.05 € kg^−1^), H_2_O_2_ (1.53 ± 0.11 € kg^−1^), and H_2_O_2_/PFA (2.88 ± 0.13 € kg^−1^) under recent and realistic assumptions (0.1723 € kWh^−1^^[^ ^47^ ^]^). These costs were ≈2–3 times higher than fossil‐based market prices for formic acid and H_2_O_2_, which already include all costs beyond electricity. Consequently, the energy demand for *eCO*_*2*_ *RR* to formate and *eO*_*2*_ *RR* to H_2_O_2_ must be reduced in the future. Nonetheless, this feasibility study demonstrates that an all‐electrochemical PFA synthesis from CO_2_, O_2_, and H_2_O is a promising approach for new sustainable chemical production processes, which should be investigated further to evaluate potential technical exploitation.

## 4 Experimental Section

4.1

4.1.1

##### Gas Diffusion Electrodes: Bi_2_O_3_‐Based GDE

The GDEs were fabricated by pressing the catalyst mixture onto Ni foam as support material. The catalyst mixture (30.00 g) consisted of Bi_2_O_3_ (87.5 wt%, 26.25 g, purity 99.9%, particle size ≈80 nm, US Research Nanomaterials, Houston, USA) and polytetrafluoroethylene (PTFE) powder (12.5 wt%, 3.75 g, Dyneon PTFE TF 2072Z, 3M, Saint Paul, USA). The mixture was homogenized in a knife mill (30 s, 25,000 rpm, 2×). Afterward, it (4.00 g) was equally distributed onto Ni foam (*d* = 1.4 cm, 3.5 cm × 4.0 cm × 14 cm^2^, Ni‐5763, density 420–450 g m^−2^, Recemat BV, Dodewaard, Netherlands) with a sieve and a stencil (cut‐out 3.5 cm × 4.0 cm). The GDE blank was placed in between ordinary baking sheet and compressed in a heat press (plate temperature 120 °C, pressure 10 bar, duration 60 s). Excess material was removed, the GDE's catalyst loading *b* was determined by differential weighing, and its thickness *d* was measured at the center point.

##### Gas Diffusion Electrodes: Carbon‐Based GDE

The GDEs were fabricated by pressing catalyst mixture consisting of carbon catalyst (65.5 wt%, 1.97 g, acetylene black, 100% compressed, >99.9%, Alfa Aesar, Haverhill, USA) and PTFE powder (34.5 wt%, 1.03 g, as above) onto a stainless steel mesh (Material 1.4301, mesh size = 0.5 cm, *d* = 240 μm, 3.2 cm × 3.2 cm, Haver & Boecker, Oelde, Germany) as support material. The catalyst mixture was homogenized (as above) and placed (500 mg) in a cylindrical mask (*d* = 40 mm) containing stainless steel mesh. The GDE blank was compressed in a hydraulic press (pressure 3.5 t, 60 s, RT followed by pressure 7 t, 180 s, RT). Afterward, the GDEs were treated in a heat press (plate temperature 120 °C, pressure 10 bar, duration 180 s) to improve their mechanical stability. Catalyst loading *b* and thickness *d* of the GDEs were determined as described above.

##### Gas Diffusion Electrodes: Scanning Electron Microscopy

SEM imaging was performed on Flex SEM 1000 II (Hitachi, Tokyo, Japan) using the following conditions: 15 kV (accelerating voltage), ×2000 (magnification), 6–8 mm (viewing height), 40 (spot size), and SE and BSE (detector). All images were taken at the GDE's geometrical center point.

##### Gas Diffusion Electrodes: Contact Angles

Contact angles were determined with the OCA 15 plus (DataPhysics Instruments, Filderstadt, Germany). Using the sessile drop method, a droplet of H_2_O (50 μL) was placed at the GDE's center point. Contact angles were calculated by fitting the droplet edges with a Young–Laplace model.

##### Gas Diffusion Electrodes: Density

GDE densities were determined with the gas pycnometer BELPYCNO L (Microtrac Retsch, Haan, Germany) using helium as probing gas. Samples were measured (*n* = 3) at 20 °C in sample chamber S (20 cm^3^) using glass beads as filler volume (≈50%).

##### Formate Electrosynthesis

The same electrochemical flow reactor (divided cell with a cation exchange membrane) and electrolysis setup as described in Dinges et al.^[^ ^9^ ^]^ were used for formate electrosynthesis.

All formate electrosyntheses were performed for 22 h at 150 mA cm^−2^ (750 mA in total) using a power supply unit (NGP804, Rohde & Schwarz, Munich, Germany), which recorded cell voltage, current, and power. The GDE's electrode potential was referenced to RHE without compensation for *iR* losses.

CO_2_ (N4.5) was supplied to the GDE at a flow rate of 10–15 mL min^−1^ and an initial overpressure in the range of 110–150 mbar relative to ambient pressure.

The buffer 0.2 mol L^−1^ KH_2_PO_4_/K_2_HPO_4_ (equimolar) served as electrolyte, both anolyte and catholyte had a starting volume of 500 mL (volumetric flask, ISO 1042). They were circulated continuously at a flow rate of ≈40 mL min^−1^ between the flow reactor and reservoir, respectively. During electrolysis, catholyte samples (1 mL) were taken hourly in the first five (*t* = 0–5 h) and the last three (*t* = 20–22 h) hours to monitor formate concentration and calculate the corresponding FE. After electrolysis, the catholyte volume was determined by its weight and density (*n* = 3). Catholyte‐containing formate was stored at 5 °C until its application for H_2_O_2_ electrosynthesis and PFA generation. The GDE was rinsed with H_2_O and dried at RT. Further details are provided (Section S1.4, Supporting Information).

##### Formate Quantification by High‐Performance Liquid Chromatography

Formate concentrations were determined via HPLC (LC‐20AD, SIL‐20AC HT, CBM‐20A, CTO‐20AC, SPD‐M20A ‐ Shimadzu, Kyoto, Japan).

The HPLC unit was equipped with a Rezex ROA‐Organic Acid (8%) column (300 mm × 7.8 mm, Phenomenex, Torrance, USA) and the following method parameters were employed: 5 mmol L^−1^ H_2_SO_4_, 0.6 mL min^−1^, 30 °C, 30 ± 1 bar, photodiode array detector (*λ* = 194 nm), 14.9 min (retention time), 25 min (duration).

Formate standards were prepared by a dilution series from a stock solution, which was prepared with HCOONa (3.482 g, 51.2 mmol) in a volumetric flask (100 mL, ISO 1042). All formate standards (8, 16, 32, 64, 128, 256, 512 mmol L^−1^) were measured (*n* = 3) and their signal areas fitted linearly (*R* ^2^ = 0.9999, fit forced through zero).

##### H_2_O_2_ Electrosynthesis for PFA Generation

The same flow reactor and electrolysis setup were used for H_2_O_2_ electrosynthesis as for formate electrosynthesis.

All electrolyses were performed for 6 h at 150 mA cm^−2^ (750 mA in total) using a power supply unit (HMC8043, Rohde & Schwarz, Munich, Germany), which recorded cell voltage and current. The GDE's electrode potential was referenced to RHE, without compensation of *iR* losses.

O_2_ (N4.6) was supplied to the GDE with a flow rate of 20 mL min^−1^ and an initial overpressure of ≈90 mbar relative to ambient pressure.

Catholyte (Formate feed) and anolyte (0.5 mol L^−1^ HClO_4_) had a starting volume of 50 mL each (measuring cylinder, 100 mL, ISO 4788) and were continuously circulated at a flow rate of ≈40 mL min^−1^ between the flow reactor and the reservoir. HClO_4_ was selected as an anolyte to ensure sufficient proton supply to the catholyte. During electrolysis, electrolytes were sampled (1 mL) every 1.5 h to determine H_2_O_2_/PFA concentrations and calculate their corresponding FE. The catholyte's volume was determined after electrolysis in the same manner as described above. Further details are provided (Section S1.5, Supporting Information).

##### H_2_O_2_/PFA Quantification by Titration

The concentrations of H_2_O_2_ and PFA were determined using a two‐step titration method derived from the procedure described by Greenspan and Mackellar.^[^ ^51^ ^]^ First, the concentration of H_2_O_2_ was determined by cerimetry using Ce(SO_4_)_2_ (*c* = 0.01 mol L^−1^). For this purpose, 5 drops of H_2_SO_4_ (*c* = 5 mol L^−1^) and 70 μL ferroin (*c* = 0.025 mol L^−1^ in ethanol) as an indicator were added to the sample solution. The orange solution was titrated until a light blue color was observed. The concentration of PFA was then determined via iodometry with Na_2_S_2_O_3_ (*c* = 0.01 mol L^−1^). To the light blue solution, 0.1 mL of a KI solution (*c* = 0.48 mol L^−1^) and a spatula tip of (NH_4_)_6_Mo_7_O_24_·4H_2_O were added. After 15 min, the resulting reddish‐brown suspension was slowly titrated until the color changed to light brown. Then 2–3 drops of starch solution (1 wt% v^−1^) were added and titration continued until the color changed back to orange and no precipitate remained. The sample volumes were 0.2 mL (after 1.5 h), 0.1 mL (after 3.0 h), and 0.075 mL (after 4.5 and 6.0 h). Each titration was carried out in triplicates (*n* = 3) for the specified time and experiment.

##### Catholyte/Feed Characterization: Ion Chromatography

IC measurements to determine K^+^ concentrations were performed on Dionex ICS‐5000^+^ DC (Pre column = Dionex IonPac CG17, Column = Dionex IonPac CS17, Analytical 2 × 250 mm, Suppressor = CERS 500, 2 mm, Thermo Fisher Scientific, Waltham, USA). Methanesulfonic acid (MSA) served as eluent with a gradient method (steps 1–4: 1. −5–0 min, 1.5 mmol L^−1^ MSA (preparation step); 2. 0–25 min, 1.5–2.1 mmol L^−1^ MSA; 3. 25–40 min, 6 mmol L^−1^ MSA; 4. 40–60 min, 1.5 mmol L^−1^ MSA) at 0.1 mL min^−1^ flow rate. Samples were diluted by factor 500 and K^+^ (retention time = 34.9 min) was detected with a conductivity cell.

Standards were prepared by a dilution series of a stock solution. The stock solution was prepared with KCl (1.221 g ≙ 640 mg K^+^) in a volumetric flask (1 L, ISO 1042). All standards (2, 4, 8, 16, 32, 64 ppm) were measured (*n* = 3) and their signal areas fitted linearly (*R* ^2^ = 0.999, fit forced through zero).

IC measurements to determine PO_4_^3−^ concentrations were performed on Dionex Aquion system (Pre column = Dionex IonPac AS22, 4 × 50 mm, Column = Dionex IonPac AS22, 4 × 250 mm, Suppressor = ACRS 500 Suppressor, 4 mm).

The following isocratic method was used: 4.5 mmol L^−1^ Na_2_CO_3_/1.4 mmol L^−1^ NaHCO_3_ (eluent), 1.2 mL min^−1^ (flow rate), 250 μL (injection volume), 15 min (duration), 9.3 min (retention time), conductivity cell (detector). Samples were diluted by factor 250.

PO_4_^3−^ standards were prepared by a dilution series from a stock solution, which was prepared from an anion multielement standard (10 mL, Certipur, Anion multielement standard I, 1000 ppm F^−^, PO_4_^3−^, Br^−^, Merck, HC17168637) in a volumetric flask (50 mL, ISO 1042).

All PO_4_^3−^ standards (25, 50, 100 mg L^−1^) were measured (*n* = 1) and their signal areas fitted linearly (*R* ^2^ = 0.999, fit forced through zero).

##### Catholyte/Feed Characterization: Inductively Coupled Plasma Optical Emission Spectroscopy

ICP‐OES measurements were performed in axial viewing mode on Agilent 5800 ICP‐OES equipped with an SPS 4 Autosampler, a borosilicate double‐pass spray chamber, and a Seaspray concentric glass nebulizer (Agilent Technologies, Santa Clara, USA).

All catholyte samples were measured without dilution except for acidification to 2 wt% HNO_3_ (using 69 wt% HNO_3_).

Standards to determine the concentrations of Bi^3+^ (*λ* = 223.061 nm), Cr^3+^ (*λ* = 205.560 nm), Fe^3+^ (*λ* = 238.204 nm), and Ni^2+^ (*λ* = 231.604 nm) were prepared by a dilution series of a stock solution (16 mg L^−1^). The stock solution was prepared by combining the respective standards (10 mL each, 1000 mg L^−1^, Single Element ICP‐Standard‐Solution, Carl Roth) in a volumetric flask (100 mL, ISO 1042) using 2 wt% HNO_3_ for dilution. Afterward, the solution was diluted further with 2 wt% HNO_3_ to 16 mg L^−1^ in a volumetric flask (50 mL, ISO 1042). This was followed by a dilution series by factor 2. Finally, each standard (0.125, 0.25, 0.5, 1, 2, 4, 8, 16 mg L^−1^) was diluted again by factor 2 with a matrix solution (0.4 mol L^−1^ KH_2_PO_4_/K_2_HPO_4_, 0.5 mol L^−1^ HCOOH, 0.5 mol L^−1^ HCOOK in 2 wt% HNO_3_). Thereby, a set of standards with a matrix based on the catholyte's composition was obtained. They were measured (*n* = 5) and their signal areas fitted linearly (*R* ^2^ ≥ 0.997, fit forced through zero). Further details are provided (Section S1.11, Supporting Information).

##### Calculations

The FE for formate and H_2_O_2_ were calculated based on the determined amount of electrosynthesized components using Equation (1).

(1) $$\text{FE}\;=\;\frac{F\cdot z\cdot n}{I\cdot t}\times 100\%$$

with *FE* = Faradaic efficiency, %; *F* = Faraday constant, A s mol^−1^; *z* = number of transferred electrons (*z* = 2); *n* = amount of synthesized formate or H_2_O_2_, mol; *I* = current, A; *t* = electrolysis runtime, s.

The results for the different catholytes were averaged and their standard deviation was provided as uncertainty.

The indirect *FE* for PFA was determined under the assumption formic acid reacted equimolar in a chemical reaction with the electrochemically generated H_2_O_2_ to PFA using Equation (2).

(2) $$H_{2}O_{2}+\text{HCOOH }⇌\text{ HCOOOH}+H_{2}O$$

The combined concentrations and *FE* of H_2_O_2_ and PFA were calculated by the addition of the individual mean values. The errors were determined using their standard deviations and Gaussian error propagation using Equation (3).
(3)

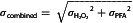




## Conflict of Interest

The authors declare no conflict of interest.

## Supporting information

Supplementary Material

## Data Availability

Data available in article supplementary material.
