# Supplementary material for: All Electrochemical Synthesis of Performic Acid Starting from CO2, O2, and H2O
Source: ChemSusChem. 2025 Apr 23;18(12):e202500180. doi: 10.1002/cssc.202500180 (PMC12175041; doi:10.1002/cssc.202500180)
Supplement: Supplementary file 1 — Supplementary Material [file CSSC-18-e202500180-s001.pdf]

Supporting Information

All Electrochemical Synthesis of Performic Acid Starting from CO_2, O_2 and H_2O

I. Dinges, M. Pyschik, J. Schütz, S. Schneider, E. Klemm, S. R. Waldvogel, M. Stöckl*

Content

1 Experimental protocols and analytical methods ... 3
1.1 General Information ... 3
1.2 Fabrication of GDE ... 5
1.3 Electrochemical flow reactor and electrolysis set-up ... 6
1.4 Electrosynthesis of formate ... 6
1.5 Electrosynthesis of H_2O_2 ... 7
1.6 Titration ... 7
1.7 X-ray diffraction (XRD) ... 8
1.8 Scanning electron microscopy (SEM) ... 8
1.9 Determination of contact angle ... 8
1.10 Determination of density ... 9
1.11 Inductively coupled plasma optical emission spectroscopy (ICP-OES) ... 10
1.12 Ion chromatography (IC) ... 14
1.13 High-performance liquid chromatography (HPLC) ... 17
2 Calculations ... 18
2.1 Faradaic efficiency (FE) ... 18
3 Results ... 19
3.1 Electrosynthesis of formate ... 19
3.2 Electrosynthesis of H_2O_2 ... 23
3.3 Pictures of GDE before and after electrolysis ... 36
3.4 SEM images of GDE before and after electrolysis ... 37
3.5 Contact angle of GDE before and after electrolysis ... 38
3.6 X-ray diffraction of GDE before and after electrolysis ... 39

1 Experimental protocols and analytical methods

1.1 General Information

Selected chemical compounds and details on employed equipment are listed in this section (cf. Table S1, Table S2). All chemicals were used without further purification and all solutions were prepared using high purity H_2O (0.055 µS cm^{-1}, 25 °C, PURELAB Ultra).

Table S1: Selected chemical compounds (Purity grade, supplier, charge number).

Compound	Purity grade	Supplier	Charge
KH_2PO_4	>99% (p.a.)	Carl Roth, Karlsruhe / Germany	453340544
K_2HPO_4	>99% (p.a.)	Carl Roth, Karlsruhe / Germany	024350089
HCOOK	99% (p.a.)	Alfa Aesar, Haverhill / USA	10183323
HCOONa	≥99% (p.a.), ACS	Merck, Darmstadt / Germany	A0703243 608
NaCl	≥99.5% (p.a.), ACS, ISO	Carl Roth, Karlsruhe / Germany	238269100
KCl	≥99.5% (p.a.), ACS, ISO	Carl Roth, Karlsruhe / Germany	392328190
H_2SO_4	75%, pure	Carl Roth, Karlsruhe / Germany	262322778
HNO_3	69%, ROTIPURAN Supra	Carl Roth, Karlsruhe / Germany	1121091
$HClO_4$	60%, EMSURE®, ACS	Merck, Darmstadt / Germany	MB2171818
KI	≥99% (p.a.)	Carl Roth, Karlsruhe / Germany	182313120
Starch solution	1%, w/v aqueous solution	Carl Roth, Karlsruhe / Germany	24011166
Ferroin solution	1/40 mol L^{-1} in ethanol	VWR, Radnor / USA	23032448
$(NH_4)_6Mo_7O_{24}\cdot 4H_2O$	≥99% (p.a.)	Carl Roth, Karlsruhe / Germany	262325940
$Na_2S_2O_3$ titrimetric solution	0.01 mol L^{-1} standard solution	Carl Roth, Karlsruhe / Germany	24022325
$Ce(SO_4)_2$ titrimetric solution	0.1 mol L^{-1} standard solution	Carl Roth, Karlsruhe / Germany	24021136
HCOOH	≥98%, for synthesis	Carl Roth, Karlsruhe / Germany	083329609
KOH	≥85%,EMSURE®, pellets for analysis	Merck, Darmstadt / Germany	B1876233
H_3PO_4	75%	VWR, Radnor / USA	17H304001

Table S2: Equipment / device, function and manufacturer.

Equipment / device	Function / use	Manufacturer
PURELAB Ultra	High purity H_2O	ELGA LabWater, High Wycombe / United Kingdom
A 10 basic	Knife mill	IKA, Staufen / Germany
LaboPress P200S	Heat press	VOGT Labormaschinen, Berlin / Germany
Perkin Elmer 062566	Hydraulic press	Bodenseewerk Perkin-Elmer, Überlingen / Germany
MicroMahr E 40	Thickness measurement	Mahr, Göttingen / Germany
Sartorius 1712004	Analytical scale (0.00000 g)	Sartorius Lab Instruments, Göttingen / Germany
Entris 3202I-1S	Scale (0.00 g)	Sartorius Lab Instruments, Göttingen / Germany
NGP804	Power supply	Rohde & Schwarz, Munich / Germany
HMC8043	Power supply	Rohde & Schwarz, Munich / Germany
CEBO-LC (CESYS C028152)	Analog data logging	CESYS, Herzogenaurach / Germany
GMH 3151	Pressure meter	GHM Messtechnik, Regenstauf / Germany
GMSD 2 BR - K31	Differential pressure sensor	GHM Messtechnik, Regenstauf / Germany
Transferpette® S	Pipetting, sampling (100-1000 µL, 500-5000 µL, 1000-10000 µL)	BRAND, Wertheim / Germany
InLab micro	pH electrode coupled with SevenCompact pH/Cond S213	Mettler Toledo, Columbus / USA
SevenCompact pH/Cond S213	pH meter	Mettler Toledo, Columbus / USA
ECOLINE VC–MS/CA8-6	Peristaltic pump	ISMATEC Laboratoriums-technik, Wertheim / Germany

1.2 Fabrication of GDE

1.2.1 GDEs for formate electrosynthesis

The gas diffusion electrodes (GDE) were fabricated by pressing a Bi_2O_3 based catalyst mixture onto Ni foam as support material and current collector with a heat press. The catalyst mixture (30.00 g) consisted of Bi_2O_3 (87.5 wt%, 26.25 g, purity 99.9%, particle size approx. 80 nm, US Research Nanomaterials, Houston / USA) and polytetrafluoroethylene (PTFE) powder (12.5 wt%, 3.75 g, Dyneon™ PTFE TF 2072Z, 3M, Saint Paul / USA). The catalyst mixture was homogenised in a knife mill (A 10 basic). The mixing (30 s, 25000 rpm) was carried out twice and lead to a temperature increase of the mixture (T >35 °C). After cooling to room temperature (RT), the catalyst mixture (4.00 g) was equally distributed onto Ni foam (d = 1.4 cm, 3.5 cm x 4.0 cm ≙ 14 cm^2, Ni-5763, density 420 - 450 g m^{-2}, Recemat BV, Dodewaard / Netherlands) with a sieve (Stainless-steel wire mesh, mesh size = 500 µm, ISO 3310-1, Retsch / Verder Scientific, Haan / Germany) and a stencil (Cut-out 3.5 cm x 4.0 cm). The GDE blank was placed in between two pieces of ordinary baking sheet in the heat press (LaboPress P200S) and compressed (plate temperature 120 °C, pressure 10 bar, 60 s). After compressing excess material at the GDE edges was removed with a scalpel. The GDE's catalyst loading *b* was determined by differential weighing (Sartorius 1712004) and its thickness *d* was measured at the centre point (Micromar 40 ER). In total, three GDEs were fabricated with a catalyst loading of *b* (Bi_2O_3, wt%) = 65.7 ± 0.7 mg cm^{-2} and *d* = 523 ± 11 µm for the electrosynthesis of formate within this study.

1.2.2 GDEs for H_2O_2 electrosynthesis

The GDEs for the (electro)synthesis of H_2O_2 and PFA were prepared by pressing a mixture containing carbon black as catalyst and PTFE as hydrophobic binder onto a stainless steel mesh as support material.

First, the catalyst mixture (3.00 g) consisting of the carbon catalyst (65.5 wt%, 1.97 g, Acetylene Black, 100% compressed, >99.9%, S.A. 75 m^2 g^{-1}, bulk density 170 - 230 g L^{-1}, Lot: Z10G042, Alfa Aesar, Haverhill / USA) and the PTFE powder (34.5 wt%, 1.03 g, Dyneon™ PTFE TF 2072Z, 3M, Saint Paul / USA) was mixed twice (each 30 s, 25000 rpm) in a knife mill (A 10 basic). Part of the mixture (500 mg) was then placed in a cylindrical mask (*d* = 40 mm) containing a stainless steel mesh (Material 1.4301, mesh size = 0.5 cm, *d* = 240 µm, 3.2 cm x 3.2 cm, Haver & Boecker, Oelde / Germany) and compressed (pressure 3.5 t, 60 s, RT followed by pressure 7 t, 180 s, RT) using a hydraulic press (Perkin Elmer 062566). After trimming the edges, the raw electrodes were treated in a heat press (plate temperature 120 °C, 10 bar, 180 s) to improve their mechanical stability.

In total, eight GDEs were fabricated with a catalyst loading of b = 26.1 ± 0.5 mg cm^{-2} and a thickness of d ≈ 600 ± 10 µm for the (electro)synthesis of H_2O_2 and PFA within this study.

1.3 Electrochemical flow reactor and electrolysis set-up

Detailed descriptions of the electrochemical flow reactor and the electrolysis set-up have already been provided in a previous publication (https://doi.org/10.1002/cssc.202301721). The same electrochemical flow reactor and electrolysis set-up have been employed for both formate and H_2O_2 electrosynthesis herein.

1.4 Electrosynthesis of formate

Electrosynthesis of formate using CO_2 was carried out with self-fabricated Bi_2O_3 based GDEs (cf. section 1.2) in a flow reactor (cf. section 1.3), whereby the GDEs were only used once per experiment.

All electrosyntheses were performed for 22 h. A power supply unit (NGP804) was employed to run the electrolysis, it recorded cell voltage (U), current (I) and power (P). Furthermore, the electrode potential of the GDE was referenced to a RHE (CEBO-LC) without compensation of iR losses. The electrosynthesis started with a current ramp (30 s), in which the current density (j) reached 150 mA cm^{-2} (750 mA in total), which was maintained for the runtime.

CO_2 (N4.5) was supplied to the GDE at a flow rate of 10 - 15 mL min^{-1} (Float-type flow meter, Wagner Mess- und Regeltechnik, Offenbach / Germany) and an initial overpressure in range of 110 - 150 mbar relative to ambient pressure. The pressure was recorded (every 2 s) during the running electrolysis (CEBO-LC).

The phosphate-based buffer 0.2 mol L^{-1} KH_2PO_4 / K_2HPO_4 (equimolar) served as electrolyte for all three electrolyses. For each electrolysis, anolyte and catholyte had a starting volume of 500 mL (Volumetric flask, ISO 1042). Anolyte and catholyte were circulated continuously at a flow rate of approx. 40 mL min^{-1} between flow reactor compartment and reservoir, respectively. Furthermore, the catholyte reservoir was equipped with a pH electrode (EGA142, Xylem Analytics Germany Sales, Weilheim / Germany). The catholyte's pH was recorded (every 2 s) during the running electrolysis (CEBO-LC). During the electrolysis, catholyte samples (1 mL, Transferpette® S) were taken hourly in the first five (t = 0 - 5 h) and the last three (t = 20 - 22 h) hours to monitor formate concentration and calculate the corresponding Faradaic efficiency (FE). After electrolysis, the catholyte volume was determined by its weight (Entris 3202I-1S) and density. Therefore, the density was calculated by taking samples (1 mL, n = 3, Transferpette® S) and weighing them (Sartorius 1712004). Catholyte containing electrochemically generated formate was stored at 5 °C until its application for (electro)synthesis of H_2O_2 and PFA. The GDE was rinsed with H_2O and dried at RT.

Detailed information on the experimental parameters and results for all individual formate electrosyntheses are provided in section 3.1.

1.5 Electrosynthesis of H_2O_2

The (electro)synthesis of H_2O_2 and PFA using O_2 was carried out with self-manufactured carbon black based GDEs in the same reactor type as the formate electrosynthesis, whereby the GDEs were only used once per experiment.

The duration of each electrolysis was 6 h. A power supply unit (HMC8043) was employed to run the electrolysis, it recorded cell voltage (U), current (I) and power (P). Furthermore, the electrode potential of the GDE was referenced to a RHE (CEBO-LC) without compensation of iR losses. The current density was 150 mA cm^{-2} (750 mA in total), which was reached by a ramp (10 s) at the beginning of the electrolysis. Catholyte and anolyte had a starting volume of 50 mL each (Measuring cylinder, 100 mL, ISO 4788) and were continuously circulated at a flow rate of approx. 40 mL min^{-1} between the flow reactor and the reservoir. Pure O_2 (N4.6) was supplied to the GDE with a flow rate of 20 mL min^{-1} and an initial overpressure of approx. 90 mbar relative to ambient pressure. For the determination of H_2O_2 / PFA concentrations and pH, 1.0 mL of the electrolytes were sampled every 1.5 h. The catholyte's volume was determined at the end of the experiments in the same manner as described above (cf. section 1.4). The catholyte did not show any gas evolution indicating H_2O_2 / PFA decomposition during or after electrolysis.

Two different phosphate-based buffers containing formate were used as catholytes: (F) The catholyte containing formate originating from formate electrosynthesis and (R) a reference electrolyte based on (F) for comparison. For all electrolyses, 0.5 mol L^{-1} perchloric acid ($HClO_4$) was used as an anolyte. Four experiments were carried out for each catholyte.

Reference electrolyte (R): 0.2 mol L^{-1} KH_2PO_4 / K_2HPO_4 (equimolar) and 0.5 mol L^{-1} HCOOK / HCOOH (equimolar) in 1 wt% H_3PO_4. The electrolyte was adjusted to pH = 4.13 ± 0.05 using KOH pellets.

1.6 Titration

The concentrations of H_2O_2 and PFA in the samples were determined using a two-step titration method similar to the procedure described by Greenspan and Mackellar (DOI: 10.1021/ac60023a020). Firstly, the concentration of H_2O_2 was determined by cerimetry using $Ce(SO_4)_2$ (c = 0.01 mol L^{-1}). For this purpose, 5 drops of H_2SO_4 (c = 5 mol L^{-1}) and 70 µL ferroin (c = 0.025 mol L^{-1} in ethanol) as an indicator were added to the sample solution. The orange solution was titrated until a light blue colour was observed. The concentration of PFA was then determined via iodometry with $Na_2S_2O_3$ (c = 0.01 mol L^{-1}). To the light blue solution, 0.1 mL of a KI solution (c = 0.48 mol L^{-1}) and a spatula tip of $(NH_4)_6Mo_7O_{24}\cdot 4H_2O$ were added.

After 15 minutes, the resulting reddish-brown suspension was slowly titrated until the colour changed to light brown. Then 2 - 3 drops of starch solution (1 wt%/v) were added and titration continued until the colour changed back to orange and no precipitates remained. The sample volumes were 0.2 mL (after 1.5 h), 0.1 mL (after 3.0 h) and 0.075 mL (after 4.5 h and 6.0 h). Each titration was carried out in triplicates ($n = 3$) for the specified time and experiment. Furthermore, results of the triplicates had statistically distributed values and did not show declining trends, which would have indicated H_2O_2 / PFA decomposition.

1.7 X-ray diffraction (XRD)

X-ray diffraction analysis was performed employing a D8 Advance XRD (Bruker, Billerica / USA), which was operated by *DIFFRAC.Measurement Center* (Version 6.5.0). Cu Kα radiation with a power of 1200 W was used, the split aperture was fixed at 2 mm, the increments were set to 0.020° and the measurement time per step was 1.0 s. XRD data was analysed with *Match!* (Version 4.0). XRD data was measured on the GDE's catalyst coated side at its geometrical centre point. Results before and after electrolysis are provided in section 3.6.

1.8 Scanning electron microscopy (SEM)

SEM imaging was performed employing the Flex SEM 1000 II (cf. Table S3), operated by *FlexSEM1000* (Version 2.3).

Table S3: Conditions for SEM imaging performed on Flex SEM 1000 II (Hitachi, Tokyo / Japan).

Conditions		Conditions	
Operation mode	Composition	Viewing height	6-8 mm
Pressure	High vacuum	Spot size	40
Accelerating voltage	15 kV	Cathode	Tungsten
Magnification	x2000, x4000	Detector	Secondary electrons (SE) and Back-scattering electrons (BSE)

Images of the GDEs were taken at the centre point of the geometrical GDE surface (2 cm x 2.5 cm) exposed within the reactor before and after application for formate and H_2O_2 / PFA (electro)synthesis. Prior to imaging, the GDEs were rinsed (H_2O) and dried (RT, 24 h). Exemplary SEM images are provided in section 3.4.

1.9 Determination of contact angle

The images for contact angle θ determination were taken with the OCA 15 plus (DataPhysics Instruments, Filderstadt / Germany), which was operated by *SCA20* (Version 4.4.1).

All measurements were carried out using the sessile drop method, a H_2O droplet (50 µL, Transferpette® S) was placed at the GDE's centre point. Contact angles were calculated by fitting the droplet edges with a Young-Laplace model using the operating software. Exemplary contact angle images are provided in section 3.5.

1.10 Determination of density

The densities of GDE were determined with the gas pycnometer BELPYCNO L (cf. Table S4), which was operated via *BELPycno-L* (Version 3.1.4).

Table S4: Volume / density measurement conditions performed on BELPYCNO L (Microtrac Retsch, Haan / Germany).

Conditions		Conditions	
Carrier gas	Helium	Restriction delta pressure	2.00000 bar
Temperature	20.00 °C	Equilibrium delta pressure	0.00020 bar
Reference volume	I	Equilibrium delta time	15 s
Flow cleaning time	0 s	Standard deviation (max.)	10%
Number of cleaning cycles	10	Nr. of good measurements	10
Sample cleaning time	5 s	Nr. of max. measurements	10
Atm. stabilisation time	15 s	High precision mode	Disabled

The reference volume chamber I was calibrated with steel calibration sphere S using the instrument's standard protocol. All samples were measured (n = 3) in sample chamber S (20 cm^3) using glass beads (⌀ = 2.85 - 3.45 mm, charge 381176662, Carl Roth, Karlsruhe / Germany) as filler volume (approx. 50%). The sample weights (Sartorius 1712004) were used to calculate the densities from the measured volumes. To measure the GDE after electrolysis, the area exposed in the reactor during electrolysis was cut out (5 cm^2). As the sample volumes were relatively small compared to the sample chamber despite the filler, the GDE for the electrosynthesis of formate and H_2O_2 were measured together in order to minimise relative errors. The results are summarised in Table S5.

Table S5: Volume and density results for Bi_2O_3 and carbon based GDE before and after electrolysis.

Sample	Electrolysis	Volume / cm^3	Density / g cm^{-3}
Bi_2O_3 based GDE	-	0.2275 ± 0.0001	7.060 ± 0.002
Bi_2O_3 based GDE	(F1), (F2), (F3)	0.3122 ± 0.0002	6.631 ± 0.004
Carbon based GDE	-	0.2634 ± 0.0003	3.094 ± 0.004
Carbon based GDE	(P1), (P2), (P3), (P4)	0.5444 ± 0.0002	3.270 ± 0.001
Carbon based GDE	(R1), (R2), (R3), (R4)	0.5144 ± 0.0002	3.363 ± 0.002

1.11 Inductively coupled plasma optical emission spectroscopy (ICP-OES)

ICP-OES measurements were performed on Agilent 5800 ICP-OES equipped with an SPS 4 Autosampler, a borosilicate double-pass spray chamber and a Seaspray concentric glass nebulizer (cf. Table S6). The system was operated via *ICP Expert* (Version 7.6.3.12735).

Table S6: ICP-OES measurement conditions performed on Agilent 5800 ICP-OES (Agilent Technologies, Santa Clara / USA).

Conditions		Conditions	
Replicate count	5	Viewing mode	Axial
Pump speed	12 rpm	Viewing height	-
Sample uptake time	25 s	Nebulizer flow	0.7 mL min^{-1}
Stabilization time	15 s	Plasma flow	12 mL min^{-1}
Read time	5 s	Aux Flow	1 mL min^{-1}
Rinse time	30 s	Oxygen content	0%
RF power	1.2 kW	IntelliQuant	Enabled

In between samples, autosampler and measurement system were rinsed with 2 wt% HNO_3 (prepared from 69 wt% HNO_3, Supra Quality, cf. Table S1).

All samples were measured without dilution except for acidification to 2 wt% HNO_3 (using 69 wt% HNO_3). In initial qualitative tests using an IntelliQuant screening, no elements of interest were detected in the anolyte samples. Consequently, only catholyte samples were examined further.

Standards to determine the concentrations of Bi^{3+} (λ = 223.061 nm), Cr^{3+} (λ = 205.560 nm), Fe^{3+} (λ = 238.204 nm) and Ni^{2+} (λ = 231.604 nm) were prepared by a dilution series of a stock solution (16 mg L^{-1}). The stock solution was prepared by combining the respective standards (10 mL each, cf. Table S7) in a volumetric flask (100 mL, ISO 1042) using 2 wt% HNO_3 for dilution. Afterwards, the solution was diluted further with 2 wt% HNO_3 to 16 mg L^{-1} in a volumetric flask (50 mL, ISO 1042). This was followed by a dilution series by factor 2. Finally, each standard (0.125, 0.25, 0.5, 1, 2, 4, 8, 16 mg L^{-1}) was diluted again by factor 2 with a matrix solution (0.4 mol L^{-1} KH_2PO_4 / K_2HPO_4, 0.5 mol L^{-1} HCOOH, 0.5 mol L^{-1} HCOOK in 2 wt% HNO_3). Thereby, a set of standards with a matrix based on the catholyte's composition was obtained. In the following, the calibrations for each targeted analyte with either catholyte matrix or 2 wt% HNO_3 matrix are presented. The calibration with catholyte matrix was used to calculate the results for the catholyte samples.

Table S7: Single Element ICP-Standard-Solutions used for analyte quantification (Element, Concentration, Supplier, Lot. No.).

Element	Concentration	Supplier	Lot. No.
Bi	1000 mg L^{-1}	Carl Roth, Karlsruhe / Germany	794591
Cr	1000 mg L^{-1}	Carl Roth, Karlsruhe / Germany	797239
Fe	1000 mg L^{-1}	Carl Roth, Karlsruhe / Germany	786848
Ni	1000 mg L^{-1}	Carl Roth, Karlsruhe / Germany	974203

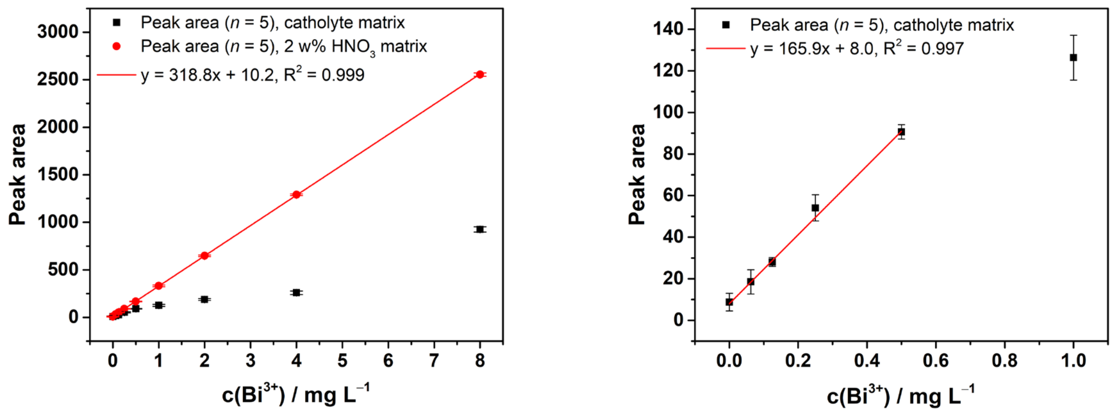

Figure S1: Calibration for Bi^{3+} (λ = 223.031 nm) via ICP-OES measurement with catholyte and 2% HNO_3 matrix**.** Plot of Bi^{3+} concentration (0, 0.0625, 0.125, 0.25, 0.5, 1, 2, 4, 8 mg L^{-1}) against the peak area of the measured signal (*n* = 5) with a linear fit, respectively.

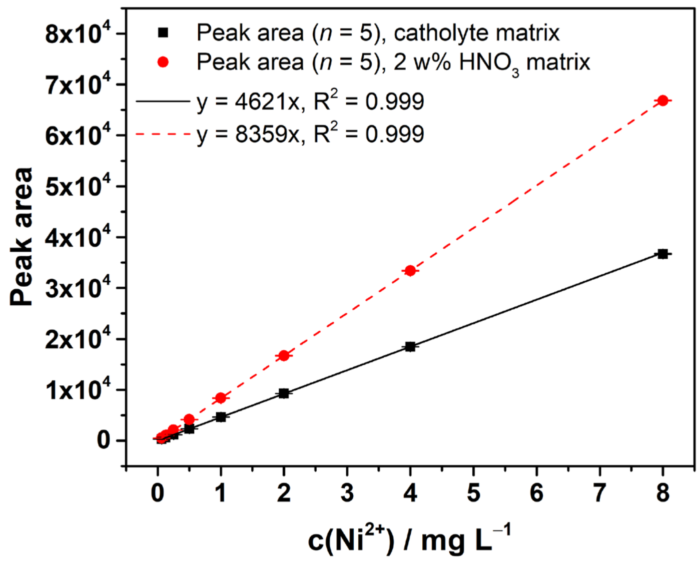

Figure S2: Calibration for Ni^{2+} (λ = 231.604 nm) via ICP-OES measurement with catholyte and 2% HNO_3 matrix**.** Plot of Ni^{2+} concentration (0.0625, 0.125, 0.25, 0.5, 1, 2, 4, 8 mg L^{-1}) against the peak area of the measured signal (*n* = 5) with a linear fit forced through zero, respectively.

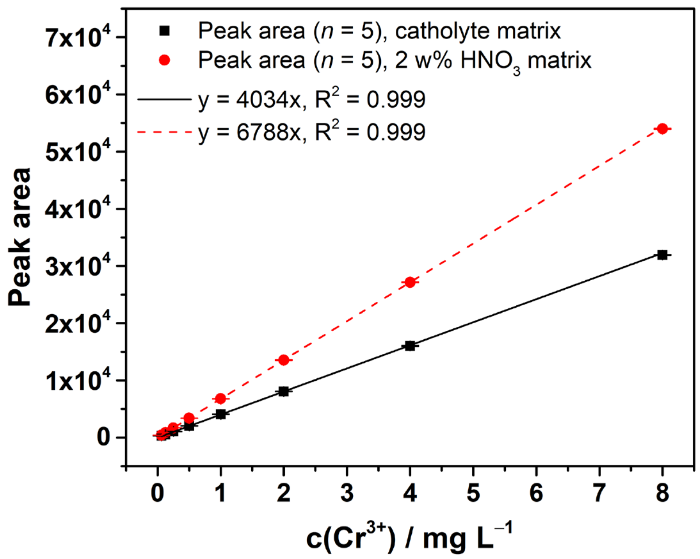

Figure S3: Calibration for Cr^{3+} (λ = 205.560 nm) via ICP-OES measurement with catholyte and 2% HNO_3 matrix**.** Plot of Cr^{3+} concentration (0.0625, 0.125, 0.25, 0.5, 1, 2, 4, 8 mg L^{-1}) against the peak area of the measured signal (*n* = 5) with a linear fit forced through zero, respectively.

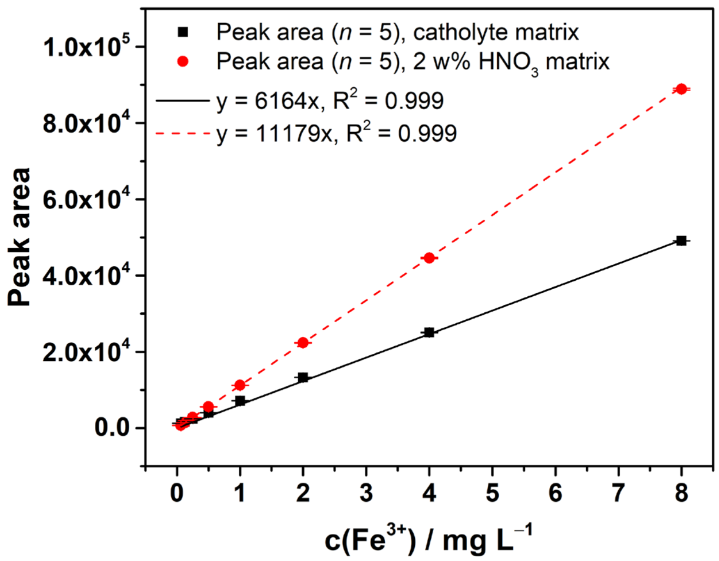

Figure S4: Calibration for Fe^{3+} (λ = 238.204 nm) via ICP-OES measurement with catholyte and 2% HNO_3 matrix**.** Plot of Fe^{3+} concentration (0.0625, 0.125, 0.25, 0.5, 1, 2, 4, 8 mg L^{-1}) against the peak area of the measured signal (*n* = 5) with a linear fit forced through zero, respectively.

Table S8: ICP-OES results for Bi^{3+}, Cr^{3+}, Fe^{3+} and Ni^{2+} concentrations in the catholyte samples.

Electrolysis	c(Bi^{3+})[c] / µg L^{-1}	c(Cr^{3+}) / µg L^{-1}	c(Fe^{3+}) / µg L^{-1}	c(Ni^{2+}) / µg L^{-1}
(F0)[a]	0	14.0 ± 0.7	151 ± 3	7.6 ± 1.9
(F1)	68 ± 25	12 ± 3	87.1 ± 0.9	19.3 ± 1.6
(F2)	98 ± 34	11.8 ± 1.7	102.6 ± 0.5	35.4 ± 1.0
(F3)	195 ± 35	11.4 ± 1.0	90.8 ± 0.6	17.1 ± 1.4
(P1)	24 ± 12	11.1 ± 2.1	99.0 ± 1.3	29.3 ± 0.6
(P2)	35 ± 20	12.6 ± 0.7	104.7 ± 1.8	49 ± 3
(P3)	54 ± 21	10.5 ± 0.9	94.3 ± 1.4	25 ± 3
(P4)	39 ± 39	14.4 ± 1.1	111.9 ± 0.7	27.1 ± 0.7
(R0)[b]	0	15.4 ± 0.5	165.7 ± 1.6	12.7 ± 1.3
(R1)	0	14.7 ± 0.7	151.2 ± 1.5	29.0 ± 1.5
(R2)	0	15.9 ± 1.2	141.2 ± 1.8	25.4 ± 1.9
(R3)	0	14.7 ± 0.4	153 ± 2	19.6 ± 1.8
(R4)	0	15.4 ± 0.5	159 ± 4	20.0 ± 0.8

[a] The phosphate buffer (0.2 mol L^{-1} KH_2PO_4 / K_2HPO_4) serving as supporting electrolyte for formate electrosynthesis was measured prior to electrolysis for comparison.

[b] The reference electrolyte (cf. section 1.5) serving as catholyte for H_2O_2 / PFA (electro)synthesis was measured prior to electrolysis for comparison.

[c] The Bi contents were below the limit of quantification (LOQ, approx. 250 µg L^{-1}) and close to the limit of detection (LOD, approx. 62.5 µg L^{-1}), which resulted in relative high standard deviations / relative errors.

1.12 Ion chromatography (IC)

IC measurements were carried out with two different IC units to determine the cation and anion concentrations in the electrolyte samples (cf. Table S9). Both units were operated with *Chromeleon* (Version 7).

Table S9: IC measurement conditions for K^+ and PO_4^{3-} analysis performed on two different IC units (Thermo Fisher Scientific, Waltham / USA).

Conditions	K^+	PO_4^{3-}
IC Unit	Dionex™ ICS-5000⁺ DC, ICS-5000⁺ SP, VWD, AS-AP autosampler	Dionex™ Aquion™ system, AS-DV autosampler
Pre column	Dionex™ IonPac™ CG17	Dionex™ IonPac™ AS22 IC column (4 x 50 mm)
Column	Dionex™ IonPac™ CS17 (Analytical 2 x 250 mm)	Dionex™ IonPac™ AS22 IC column (4 x 250 mm)
Suppressor	CERS 500 (2 mm)	ACRS 500 Suppressor (4 mm)
Eluent	6 mmol L^{-1} CH_3SO_3H (MSA)	4.5 mmol L^{-1} Na_2CO_3 / 1.4 mmol L^{-1} $NaHCO_3$
Flow rate	0.1 mL min^{-1}	1.2 mL min^{-1}
Method	gradient<br>1. −5 - 0 min, 1.5 mmol L^{-1} MSA<br>2. 0 - 25 min, 1.5 - 2.1 mmol L^{-1} MSA<br>3. 25 - 40 min, 6 mmol L^{-1} MSA<br>4. 40 - 60 min, 1.5 mmol L^{-1} MSA	isocratic
Injection volume	10 µL	250 µL
Sample dilution factor	500	250
Detector	Conductivity cell	Conductivity cell
Retention time	34.87 min	9.28 min
Duration	60 min	15 min

Samples were diluted by factor 250 in a volumetric flask (50 mL, ISO 1042) for PO_4^{3-} quantification. The samples were diluted further by factor 2 for K^+ quantification.

Standards to determine the concentrations of K^+ were prepared by a dilution series of a stock solution. The stock solution was prepared with KCl (1.221 g ≙ 640 ppm K^+) in a volumetric

flask (1 L, ISO 1042). The stock solution was first diluted by 10, followed by a dilution series with the dilution factor 2 (cf. Figure S5).

The PO_4^{3-} standards were prepared from an anion multi-element standard (Certipur, Anion multi-element standard I, 1000 ppm F^-, PO_4^{3-}, Br^-, Merck, Darmstadt / Germany, HC17168637) by a dilution series (cf. Figure S6).

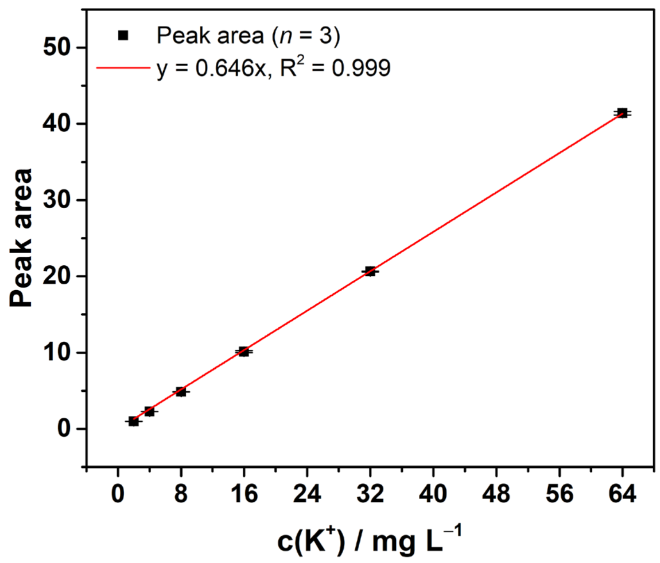

Figure S5: Calibration for K^+ quantification via IC measurement**.** Plot of K^+ concentration (2, 4, 8, 16, 32, 64 ppm) against the peak area of the measured signal (n = 3) with a linear fit forced through zero.

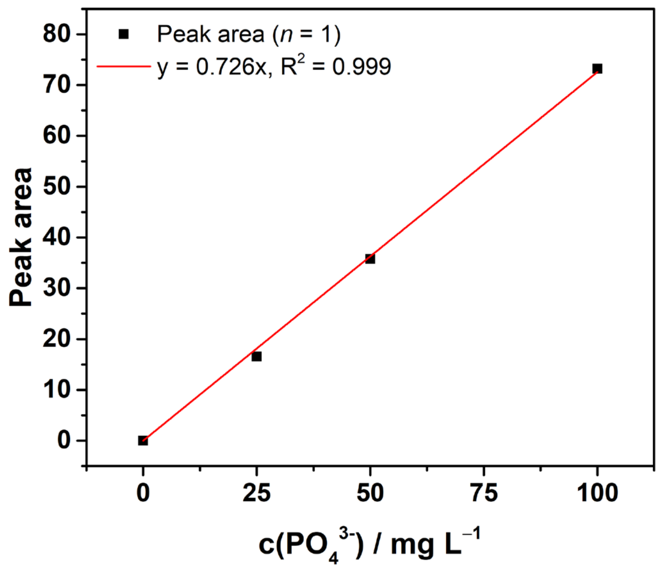

Figure S6: Calibration for PO_4^{3-} quantification via IC measurement**.** Plot of PO_4^{3-} concentration (0, 25, 50, 100 ppm) against the peak area of the measured signal (n = 1) with a linear fit forced through zero.

Table S10: Concentrations of K^+ and PO_4^{3-} determined by IC in catholyte and anolyte after electrolysis (6 or 22 h, 150 mA cm^{-2}) with different catholytes: 0.2 mol L^{-1} KH_2PO_4 / K_2HPO_4, (F) Combined catholytes containing formate, (R) Reference electrolyte (cf. section 1.5).

Electrolysis	c(K^+) / mmol L^{-1}		c(PO_4^{3-}) / mmol L^{-1}	
	Catholyte	Anolyte	Catholyte	Anolyte
(F0)[a]	286.2	286.2	197.5	197.5
(F1)	548.0	3.0	183.6	209.8
(F2)	540.4	2.7	183.5	210.3
(F3)	537.9	3.4	183.0	206.1
(P1)	383.4	13.2	133.5	0.8
(P2)	448.0	14.8	154.7	0.3
(P3)	433.9	11.6	150.8	0.4
(P4)	425.8	10.9	148.2	0.4
(R0)[b]	672.2	-	288.6	-
(R1)	533.2	12.9	238.0	0.4
(R2)	550.5	12.8	245.2	0.4
(R3)	532.5	12.2	236.1	0.4
(R4)	529.3	13.2	237.7	0.3

[a] The phosphate buffer (0.2 mol L^{-1} KH_2PO_4 / K_2HPO_4) serving as supporting electrolyte for formate electrosynthesis was measured prior to electrolysis for comparison.

[b] The reference electrolyte (R, cf. section 1.5) serving as catholyte for H_2O_2 / PFA (electro)synthesis was measured prior to electrolysis for comparison.

1.13 High-performance liquid chromatography (HPLC)

The quantification of formate was carried out via HPLC (cf. Table S11), the system was operated with the software *LabSolutions* (Version 5.93).

Table S11: HPLC measurement conditions for formate and PHB analysis performed on an HPLC unit (LC-20AD, SIL-20AC HT, CBM-20A, CTO-20AC, SPD-M20A - Shimadzu, Kyoto / Japan).

Conditions	Formate
Eluent	5 mmol L^{-1} H_2SO_4
Flow rate	0.6 mL min^{-1}
Pressure	30 ± 1
Column oven	35 °C
Column	Rezex ROA- Organic Acid (8%), 300 mm × 7.8 mm, Phenomenex, Torrance / USA
Injection volume	10 µL
Detector	Photodiode array (PDA)
Wavelength λ	194 nm
Retention time	14.9 min
Duration	25 min

Formate standards were prepared from a stock solution by a dilution series with the dilution factor 2. The stock solution was prepared with HCOONa (3.482 g, 51.2 mmol) in a volumetric flask (100 mL, ISO 1042).

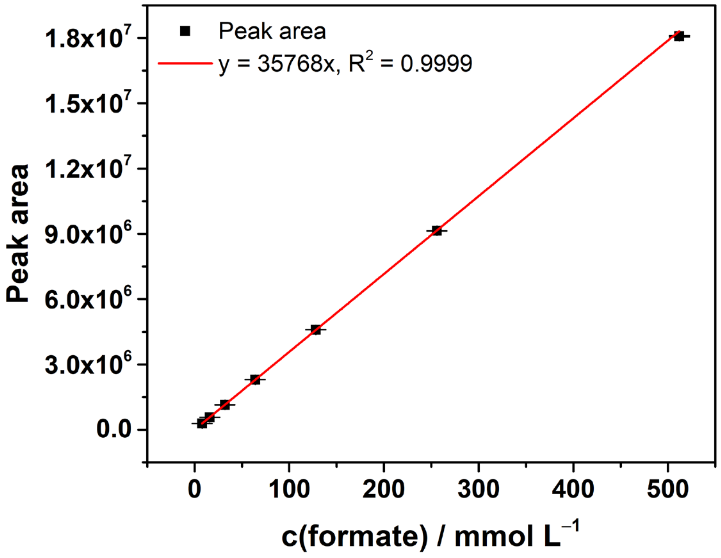

Figure S7: Calibration for formate quantification via HPLC measurement. Plot of formate concentration (8, 16, 32, 64, 128, 256, 512 mmol L^{-1}) against the peak area of the measured signal (n = 3) with a linear fit forced through zero.

2 Calculations

2.1 Faradaic efficiency (FE)

The FE for formate and H_2O_2 were calculated based on the determined amount of electrosynthesised components using equation (1).

$$FE = \frac{F \cdot z \cdot n}{I \cdot t} \cdot 100\% \quad (1)$$

With FE = Faradaic efficiency / %, F = Faraday constant / A s mol^{-1}, z = Number of transferred electrons (z = 2), n = Amount of synthesised formate or H_2O_2 / mol, I = Current / A, t = Electrolysis runtime / s.

The results for the different catholytes were averaged and their standard deviation was provided as uncertainty.

The indirect FE for PFA was determined under the assumption that formic acid reacted equimolar in a chemical reaction with the electrochemically generated H_2O_2 to PFA using equation (2).

$$H_2O_2 + HCOOH \rightleftharpoons HCOOOH + H_2O \quad (2)$$

The combined concentrations and FE of H_2O_2 and PFA were calculated by addition of the individual mean values. The errors were determined using their standard deviations and Gaussian error propagation using equation (3).

$$\sigma_{\mathrm{combined}} = \sqrt{{\sigma_{\mathrm{H_2O_2}}}^2 + {\sigma_{\mathrm{PFA}}}^2} \quad (3)$$

3 Results

This chapter contains detailed data for formate electrosynthesis in section 3.1 and for H_2O_2 / PFA electro(synthesis) in section 3.2. Furthermore, GDE photographs are provided in section 3.3, which is followed by SEM images in section 3.4 and contact angle photographs in section 3.5 of the self-fabricated GDEs before and after electrolysis, respectively.

3.1 Electrosynthesis of formate

The operational electrolysis parameters have been summarised in Table S12, further electrolysis results have been collected in Table S13. Moreover, detailed courses of each individual electrolysis are provided.

Table S12: Overview of operational electrolysis parameters of the formate electrosynthesis using 0.2 mol L^{-1} KH_2PO_4 / K_2HPO_4 as electrolyte. All values are given as average with standard deviation for the 22 h electrolysis duration, excluding the absolute electric energy consumption (EEC).

Electrolysis	OCP vs. RHE / V[a]	$E(GDE)$ vs. RHE / V	U / V	EEC / Wh	$p(CO_2)$ / mbar
(F1)	0.67 ± 0.03	−1.25 ± 0.12	5.6 ± 0.4	92.2	292 ± 29
(F2)	0.273 ± 0.002	−1.20 ± 0.14	5.9 ± 0.4	96.9	263 ± 44
(F3)	0.268 ± 0.004	−1.19 ± 0.10	6.0 ± 0.5	98.9	272 ± 33

[a] Average with standard deviation measured for 5 min prior to electrolysis.

Table S13: Overview of volume, formate concentration, formate FE and pH determined *n* = 3 after 22 h electrolysis in the catholyte of formate electrosynthesis with 0.2 mol L^{-1} KH_2PO_4 / K_2HPO_4 as starting electrolyte.

Electrolysis	V / mL	c(formate) / mmol L^{-1}	FE(formate) / %	pH
(F1)	529.4 ± 0.6	501.6 ± 0.3	86.34 ± 0.06	4.12 ± 0.05
(F2)	531.5 ± 0.7	501.0 ± 0.4	86.50 ± 0.13	4.12 ± 0.05
(F3)	529.5 ± 0.7	499.8 ± 0.6	86.0 ± 0.3	4.10 ± 0.05

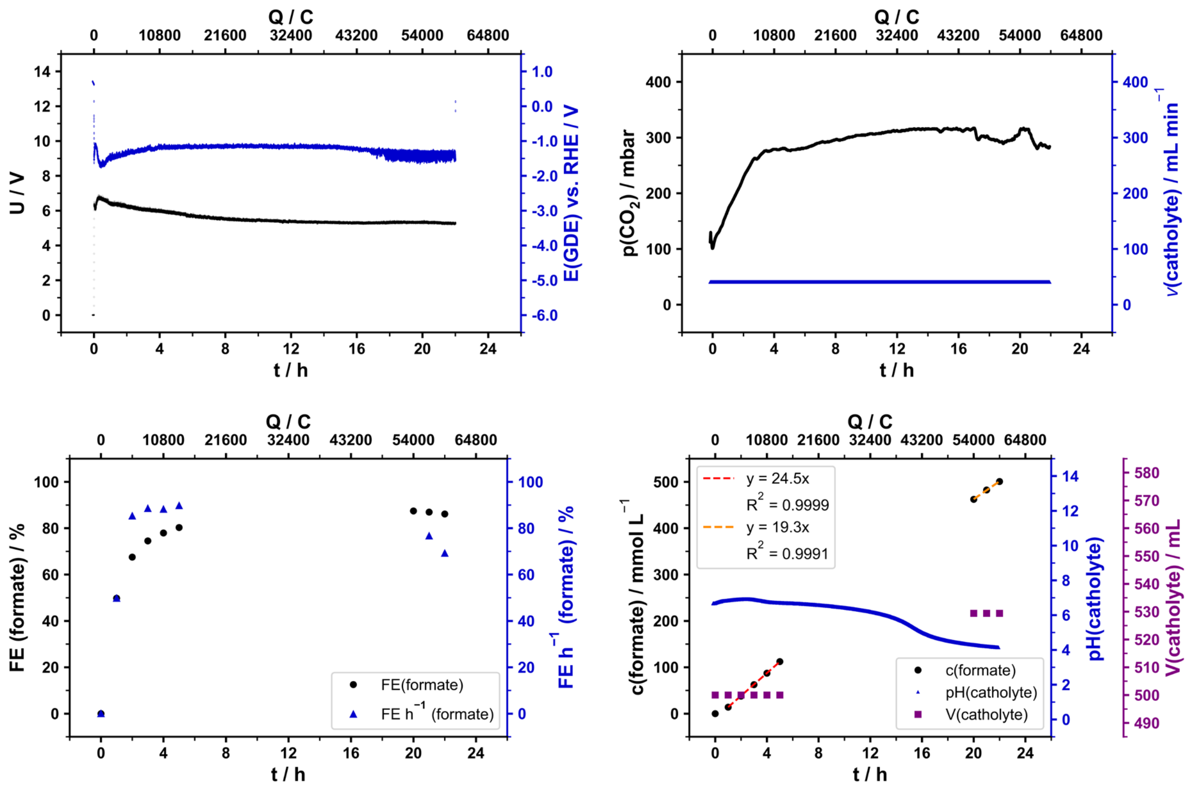

Figure S8: Data for Electrolysis (F1), experimental details are provided in section 1.4 and results in Table S12 and Table S13.

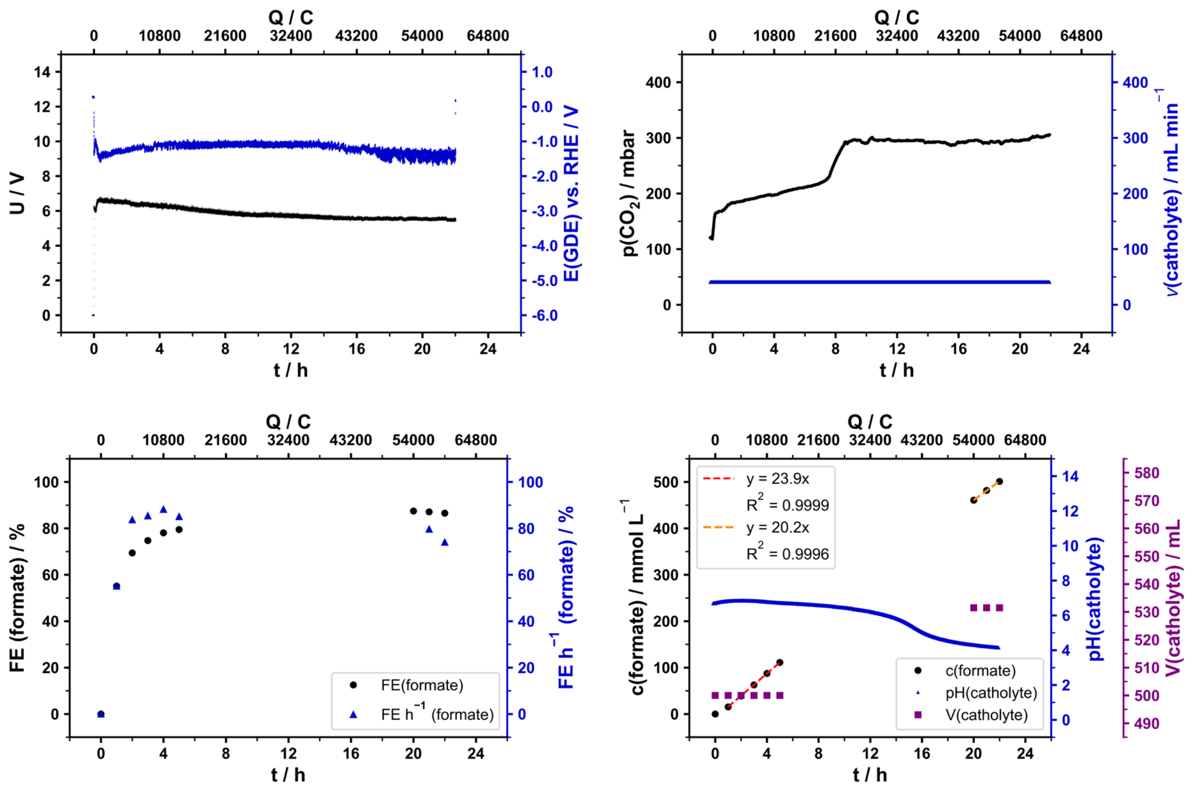

Figure S9: Data for Electrolysis (F2), experimental details are provided in section 1.4 and results in Table S12 and Table S13.

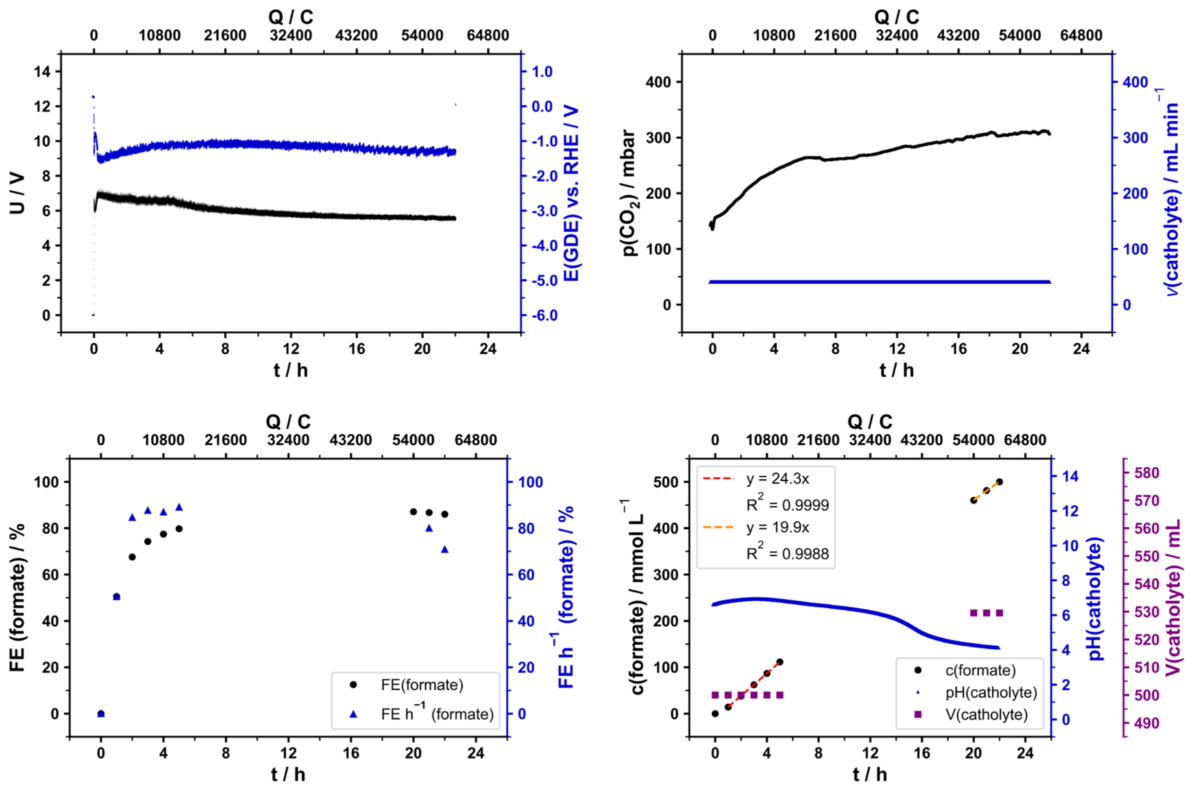

Figure S10: Data for Electrolysis (F3), experimental details are provided in section 1.4 and results in Table S12 and Table S13.

3.2 Electrosynthesis of H_2O_2

The operational electrolysis parameters have been summarised in Table S14, further electrolysis results have been collected in Table S15. Moreover, detailed courses of each individual electrolysis are provided.

Table S14: Overview of operational electrolysis parameters of the H_2O_2 / PFA (electro)synthesis using (F) or (R) as starting electrolyte (cf. section 1.5). All values are given as average with standard deviation for the 6 h electrolysis duration, excluding the absolute electric energy consumption (EEC).

Electrolysis	$E(GDE)$ vs. RHE / V	U / V	EEC / Wh	$p(O_2)$ / mbar
(P1)	−1.75 ± 0.36	4.7 ± 0.2	21.2	111 ± 22
(P2)	−0.68 ± 0.23	4.7 ± 0.2	21.2	121 ± 18
(P3)	−1.52 ± 0.32	4.8 ± 0.2	21.6	132 ± 38
(P4)	−1.59 ± 0.62	4.9 ± 0.2	22.1	146 ± 44
(R1)	−1.75 ± 0.30	4.7 ± 0.1	21.2	184 ± 33
(R2)	−1.44 ± 0.29	4.8 ± 0.1	21.6	193 ± 33
(R3)	−1.40 ± 0.33	4.4 ± 0.2	19.8	210 ± 56
(R4)	−1.40 ± 0.32	4.7 ± 0.1	21.2	174 ± 50

Table S15: Overview of volume, H_2O_2 / PFA concentrations, H_2O_2 / PFA FE and pH determined n = 3 after 6 h electrolysis in the catholyte of H_2O_2 / PFA (electro)synthesis with (F) or (R) as starting electrolyte (cf. section 1.5).

Electrolysis	V / mL	c(H_2O_2) / mol L^{-1}	FE(H_2O_2) / %	c(PFA) / mmol L^{-1}	FE(PFA) / %	pH
(P1)	52.3 ± 0.1	1.1855 ± 0.0051	73.8 ± 0.4	70.7 ± 2.7	4.4 ± 0.2	3.92 ± 0.05
(P2)	53.1 ± 0.2	1.3700 ± 0.0122	86.6 ± 0.8	78.7 ± 11.4	5.0 ± 0.8	3.93 ± 0.05
(P3)	54.1 ± 0.2	1.2600 ± 0.0034	81.2 ± 0.3	81.3 ± 4.7	5.2 ± 0.3	3.93 ± 0.05
(P4)	52.0 ± 0.2	1.2756 ± 0.0051	79.0 ± 0.4	96.4 ± 2.8	6.0 ± 0.2	3.92 ± 0.05
(R1)	54.0 ± 0.2	1.2389 ± 0.0084	79.6 ± 0.6	69.3 ± 4.7	4.5 ± 0.5	3.94 ± 0.05
(R2)	54.3 ± 0.3	1.0311 ± 0.0070	66.7 ± 0.5	64.4 ± 3.9	4.2 ± 0.3	3.96 ± 0.05
(R3)	54.8 ± 0.3	1.1722 ± 0.0051	76.5 ± 0.4	72.9 ± 0.8	4.8 ± 0.1	3.98 ± 0.05
(R4)	54.4 ± 0.2	1.0833 ± 0.0034	70.2 ± 0.3	67.6 ± 0.8	4.4 ± 0.1	4.00 ± 0.05

3.2.1 Catholyte (F) containing formate from formate electrosynthesis

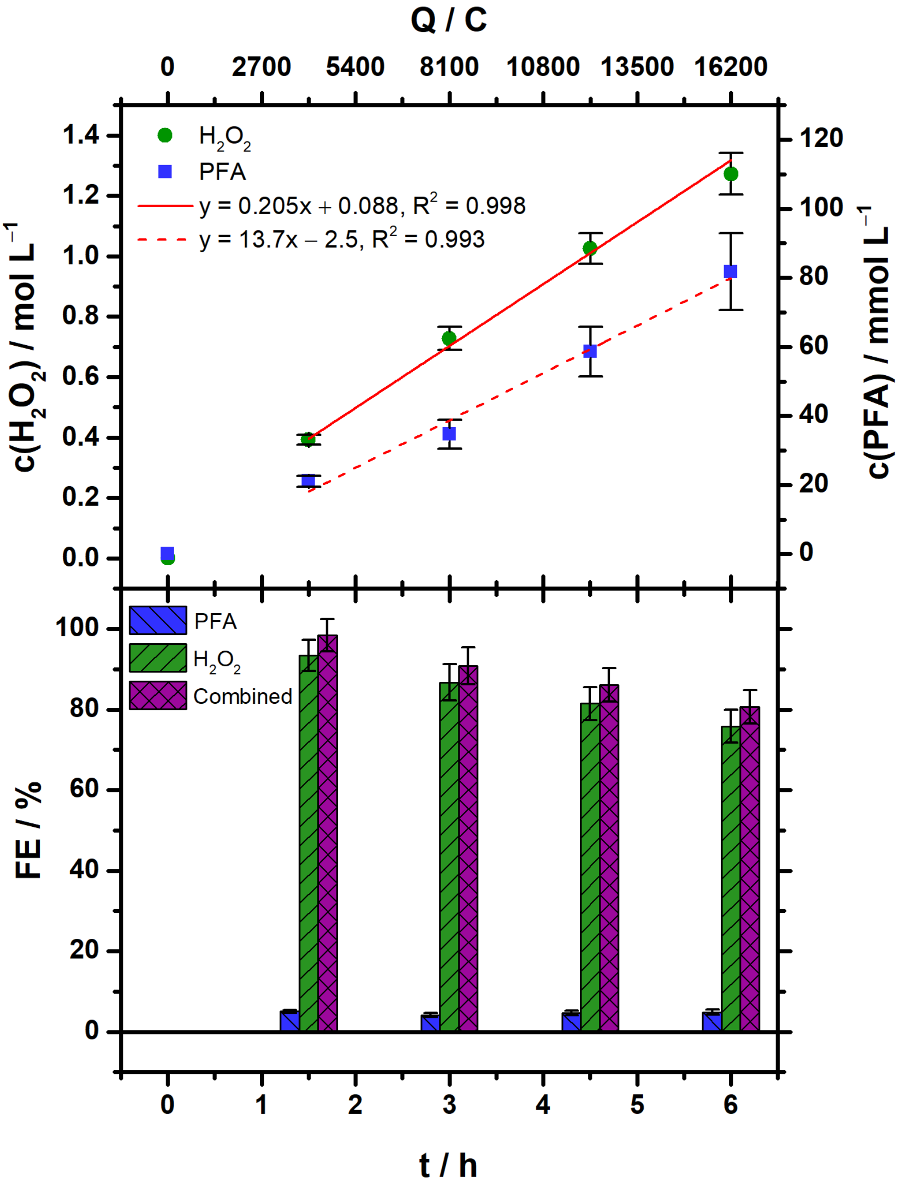

Figure S11: Concentration and Faradaic efficiency (FE) course of H_2O_2 and PFA for H_2O_2 / PFA (electro)synthesis (n = 4). Electrolysis parameters: Constant current density j = 150 mA cm^{-2}, Runtime = 6 h (≙ 16200 C), Catholyte = Formate containing catholyte originating from formate electrosynthesis (50 mL), Anolyte = 0.5 mol L^{-1} $HClO_4$ (50 mL), Cathode (GDE) = 65.5 wt% Acetylene Black, 34.5 wt% PTFE on stainless steel mesh, Reference electrode = Reversible hydrogen electrode (RHE), Anode = Mixed Ir-oxide on a Ti-grid (Platinode EP, Type 177, Umicore).

Table S16: Concentration and Faradaic efficiency (FE) of H_2O_2 and PFA in the catholyte for electrolysis (P1) with 0.2 mol L^{-1} KH_2PO_4 / K_2HPO_4 containing formate originating from formate electrosynthesis as starting electrolyte (cf. Figure S12).

t / h	$c(H_2O_2)$ / mol L^{-1}	$FE(H_2O_2)$ / %	c(PFA) / mmol L^{-1}	FE(PFA) / %
1.5	0.3729 ± 0.0008	88.8 ± 0.2	23.0 ± 0.5	5.5 ± 0.2
3	0.6758 ± 0.0029	80.5 ± 0.4	31.7 ± 0.5	3.8 ± 0.5
4.5	0.9544 ± 0.0020	75.8 ± 0.2	64.0 ± 1.1	5.1 ± 0.9
6	1.1855 ± 0.0051	70.6 ± 0.3	70.7 ± 2.7	4.2 ± 0.2

Table S17: pH for electrolysis (P1) with 0.2 mol L^{-1} KH_2PO_4 / K_2HPO_4 containing formate originating from formate electrosynthesis as starting electrolyte.

t / h	pH (catholyte)	pH (anolyte)
0	4.11 ± 0.05	0.39 ± 0.05
1.5	3.92 ± 0.05	0.38 ± 0.05
3	3.93 ± 0.05	0.38 ± 0.05
4.5	3.93 ± 0.05	0.33 ± 0.05
6	3.92 ± 0.05	0.34 ± 0.05

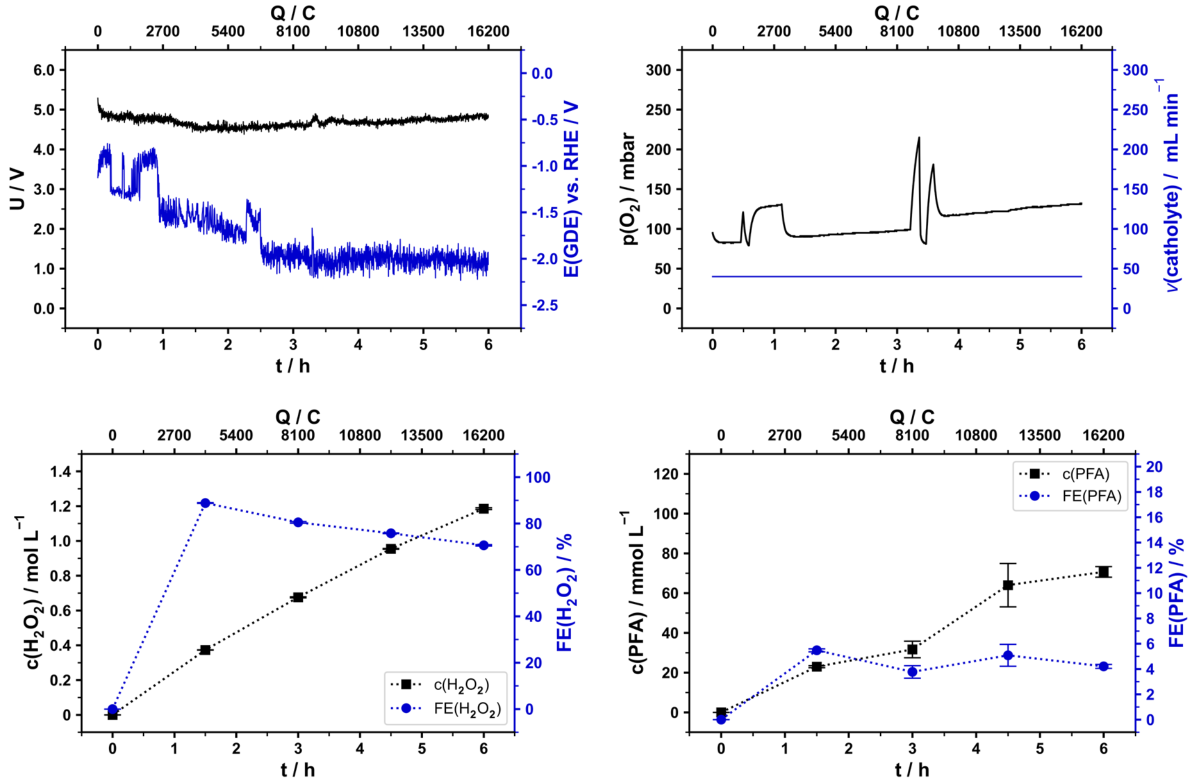

Figure S12: Data for electrolysis (P1), experimental details are provided in section 1.5 and results in Table S14 and Table S15.

Table S18: Concentration and Faradaic efficiency (FE) of H_2O_2 and PFA in the catholyte for electrolysis (P2) with 0.2 mol L^{-1} KH_2PO_4 / K_2HPO_4 containing formate originating from formate electrosynthesis as starting electrolyte (cf. Figure S13).

t / h	c(H_2O_2) / mol L^{-1}	FE(H_2O_2) / %	c(PFA) / mmol L^{-1}	FE(PFA) / %
1.5	0.4158 ± 0.0015	99.1 ± 0.4	19.8 ± 0.3	4.7 ± 0.1
3	0.7775 ± 0.0025	92.6 ± 0.3	34.7 ± 1.2	4.1 ± 0.2
4.5	1.0911 ± 0.0051	86.6 ± 0.5	56.9 ± 8.8	4.5 ± 0.7
6	1.3700 ± 0.0122	81.6 ± 0.8	78.7 ± 11.4	4.7 ± 0.7

Table S19: pH for electrolysis (P2) with 0.2 mol L^{-1} KH_2PO_4 / K_2HPO_4 containing formate originating from formate electrosynthesis as starting electrolyte.

t / h	pH (catholyte)	pH (anolyte)
0	4.10	0.38
1.5	3.93	0.29
3	3.93	0.30
4.5	3.92	0.27
6	3.93	0.23

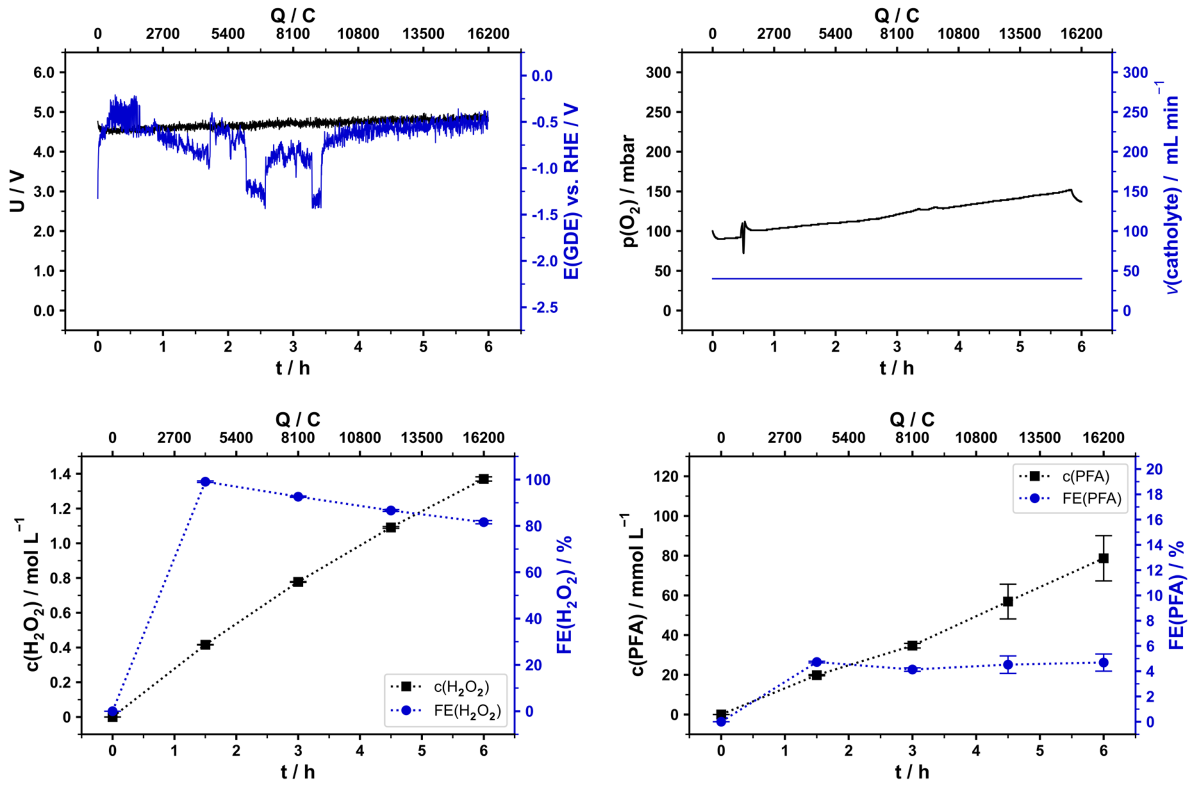

Figure S13: Data for electrolysis (P2), experimental details are provided in section 1.5 and results in Table S14 and Table S15.

Table S20: Concentration and Faradaic efficiency (FE) of H_2O_2 and PFA in the catholyte for electrolysis (P3) with 0.2 mol L^{-1} KH_2PO_4 / K_2HPO_4 containing formate originating from formate electrosynthesis as starting electrolyte (cf. Figure S14).

t / h	$c(H_2O_2)$ / mol L^{-1}	$FE(H_2O_2)$ / %	c(PFA) / mmol L^{-1}	FE(PFA) / %
1.5	0.3846 ± 0.0020	91.6 ± 0.5	19.8 ± 0.2	4.7 ± 0.3
3	0.7217 ± 0.0015	86.0 ± 0.2	32.0 ± 1.0	3.8 ± 0.2
4.5	1.0189 ± 0.0020	80.9 ± 0.2	55.6 ± 4.3	4.4 ± 0.4
6	1.2600 ± 0.0034	75.0 ± 0.2	81.3 ± 4.7	4.8 ± 0.3

Table S21: pH for electrolysis (P3) with 0.2 mol L^{-1} KH_2PO_4 / K_2HPO_4 containing formate originating from formate electrosynthesis as starting electrolyte.

t / h	pH (catholyte)	pH (anolyte)
0	4.00	0.33
1.5	3.96	0.33
3	3.95	0.32
4.5	3.94	0.28
6	3.93	0.24

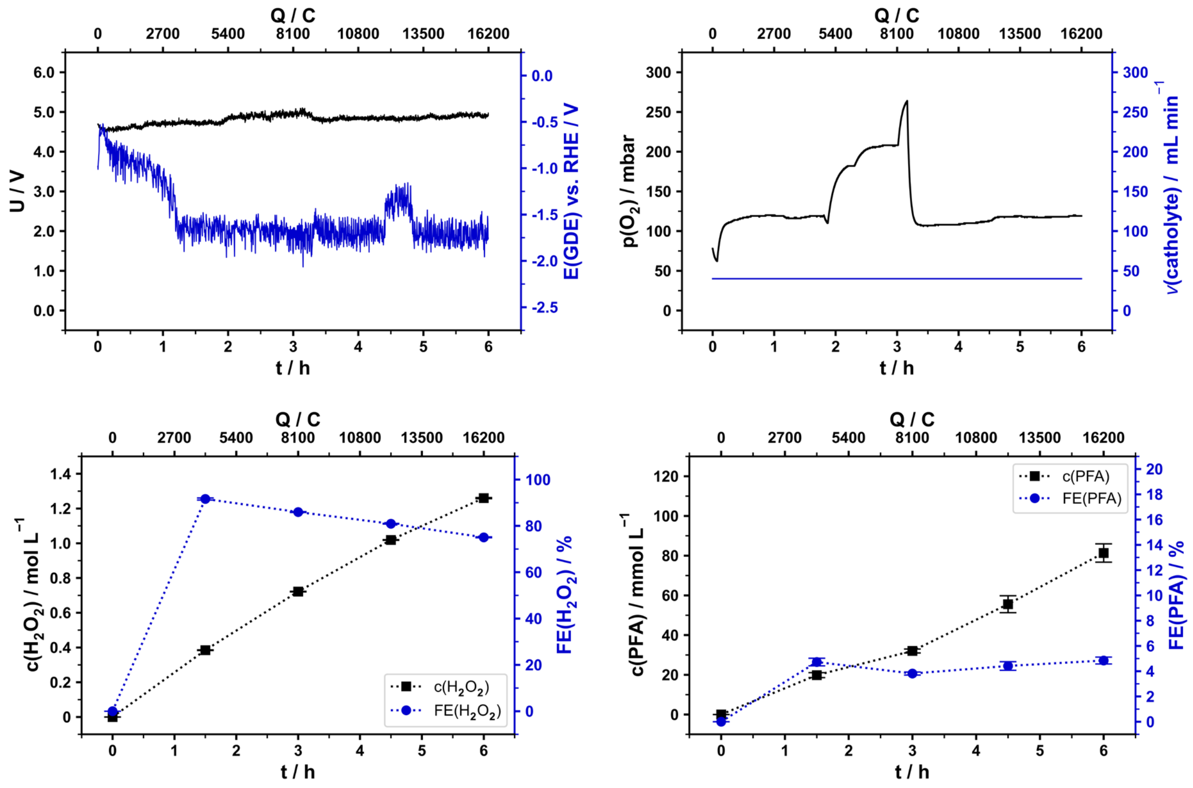

Figure S14: Data for electrolysis (P3), experimental details are provided in section 1.5 and results in Table S14 and Table S15.

Table S22: Concentration and Faradaic efficiency (FE) of H_2O_2 and PFA in the catholyte for electrolysis (P4) with 0.2 mol L^{-1} KH_2PO_4 / K_2HPO_4 containing formate originating from formate electrosynthesis as starting electrolyte (cf. Figure S15).

t / h	$c(H_2O_2)$ / mol L^{-1}	$FE(H_2O_2)$ / %	c(PFA) / mmol L^{-1}	FE(PFA) / %
1.5	0.3950 ± 0.0013	94.1 ± 0.3	21.7 ± 1.2	5.2 ± 0.3
3	0.7367 ± 0.0015	87.8 ± 0.2	40.3 ± 2.1	4.8 ± 0.3
4.5	1.0378 ± 0.0020	82.4 ± 0.2	58.2 ± 2.8	4.6 ± 0.3
6	1.2756 ± 0.0051	76.0 ± 0.4	96.4 ± 2.8	5.7 ± 0.2

Table S23: pH for electrolysis (P4) with 0.2 mol L^{-1} KH_2PO_4 / K_2HPO_4 containing formate originating from formate electrosynthesis as starting electrolyte.

t / h	pH (catholyte)	pH (anolyte)
0	3.99	0.34
1.5	3.95	0.34
3	3.94	0.34
4.5	3.94	0.30
6	3.92	0.27

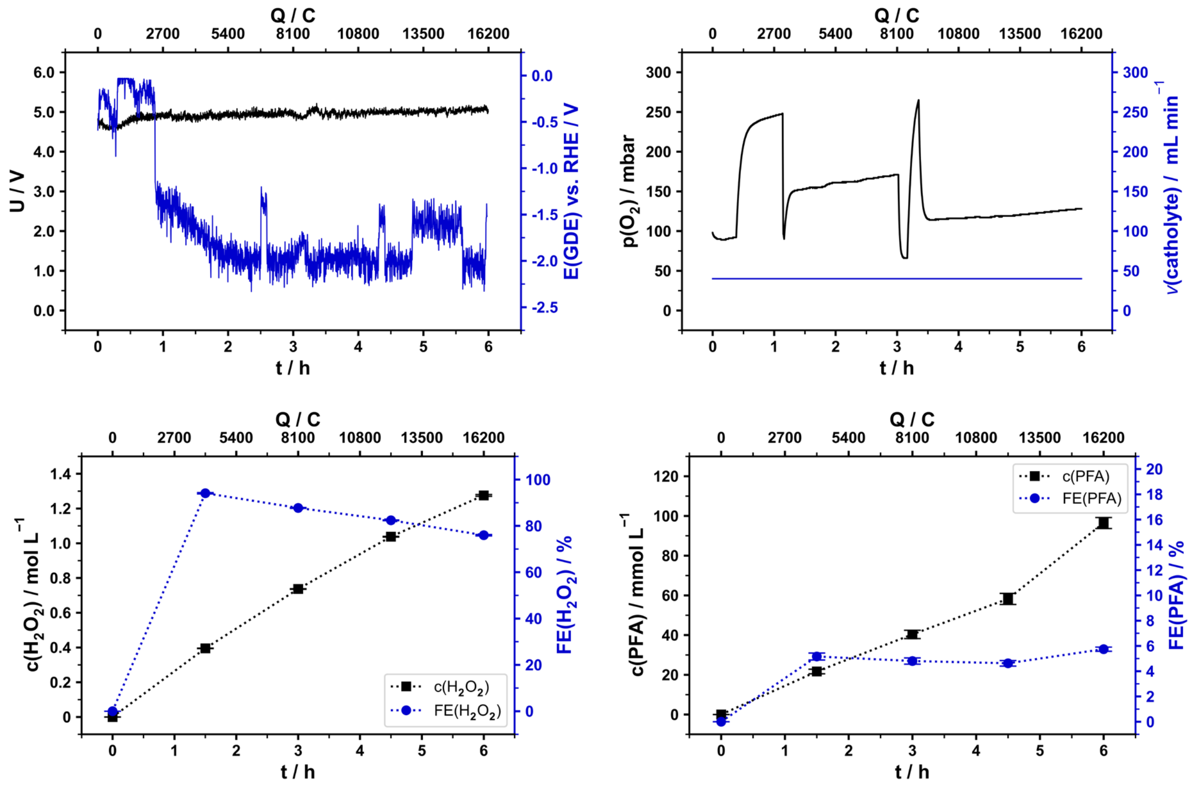

Figure S15: Data for electrolysis (P4), experimental details are provided in section 1.5 and results in Table S14 and Table S15.

3.2.2 Reference electrolyte (R) containing 0.5 mol L^{-1} HCOOK / HCOOH as catholyte

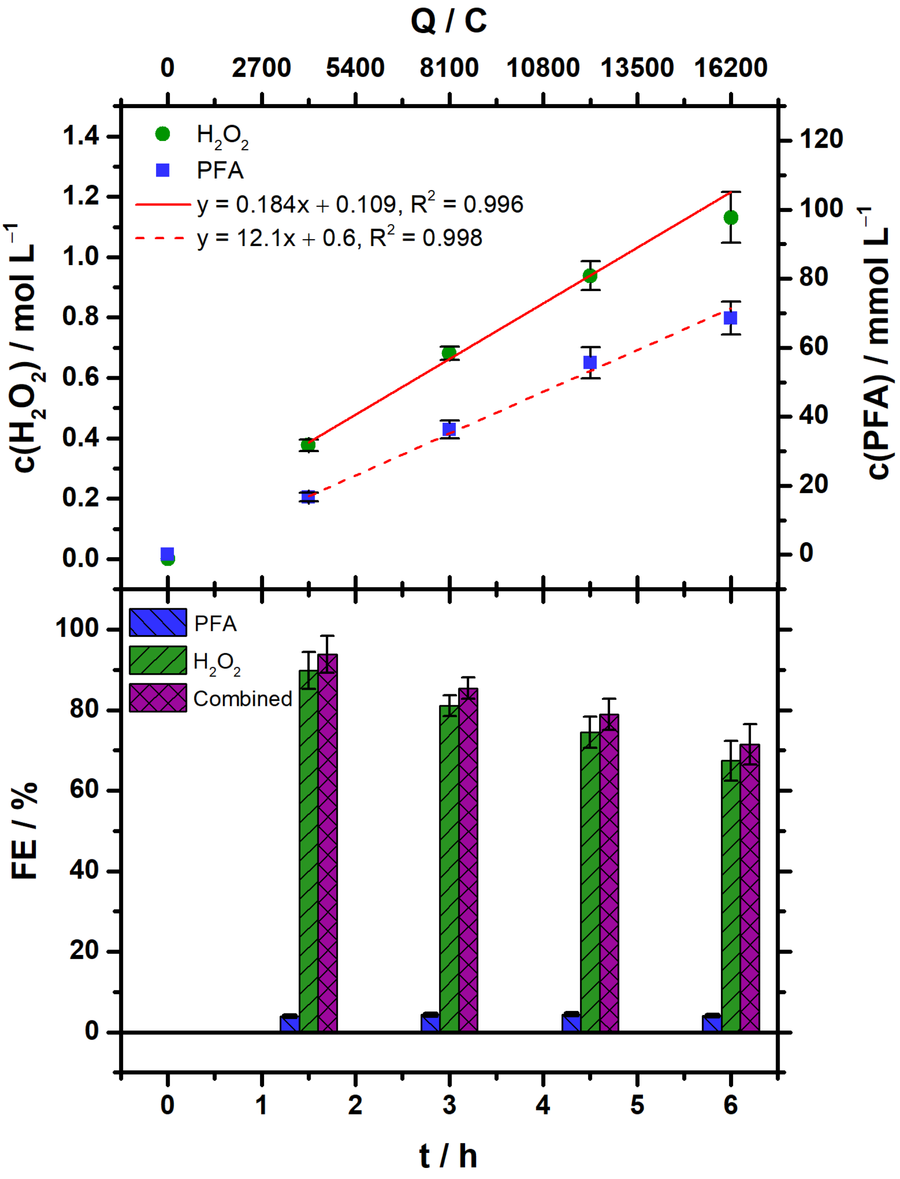

Figure S16: Concentration and Faradaic efficiency (FE) course of H_2O_2 and PFA for H_2O_2 / PFA (electro)synthesis ($n = 4$). Electrolysis parameters: Constant current density j = 150 mA cm^{-2}, Runtime = 6 h (≙ 16200 C), Catholyte = 0.2 mol L^{-1} KH_2PO_4 / K_2HPO_4 (equimolar) and 0.5 mol L^{-1} HCOOK / HCOOH (equimolar) in 1 wt% H_3PO_4 (50 mL, reference electrolyte (R), cf. section 1.5), Anolyte = 0.5 mol L^{-1} $HClO_4$ (50 mL), Cathode (GDE) = 65.5 wt% Acetylene Black, 34.5 wt% PTFE on stainless steel mesh, Reference electrode = Reversible hydrogen electrode (RHE), Anode = Mixed Ir-oxide on a Ti-grid (Platinode EP, Type 177, Umicore).

Table S24: Concentration and Faradaic efficiency (FE) of H_2O_2 and PFA in the catholyte for electrolysis (R1) with 0.2 mol L^{-1} KH_2PO_4 / K_2HPO_4 containing 0.5 mol L^{-1} HCOOK / HCOOH in 1 wt% H_3PO_4 as starting electrolyte (cf. Figure S17).

t / h	c(H_2O_2) / mol L^{-1}	FE(H_2O_2) / %	c(PFA) / mmol L^{-1}	FE(PFA) / %
1.5	0.4067 ± 0.0060	96.9 ± 1.5	17.0 ± 2.0	4.1 ± 0.5
3	0.7142 ± 0.0063	85.1 ± 0.8	35.3 ± 2.1	4.2 ± 0.3
4.5	1.0333 ± 0.0034	79.7 ± 0.3	51.1 ± 2.1	4.1 ± 0.2
6	1.2389 ± 0.0084	73.8 ± 0.5	69.3 ± 4.7	5.1 ± 0.3

Table S25: pH for electrolysis (R1) with 0.2 mol L^{-1} KH_2PO_4 / K_2HPO_4 containing 0.5 mol L^{-1} HCOOK / HCOOH in 1 wt% H_3PO_4 as starting electrolyte.

t / h	pH (catholyte)	pH (anolyte)
0	4.07	0.35
1.5	3.96	0.32
3	3.94	0.29
4.5	3.93	0.28
6	3.94	0.27

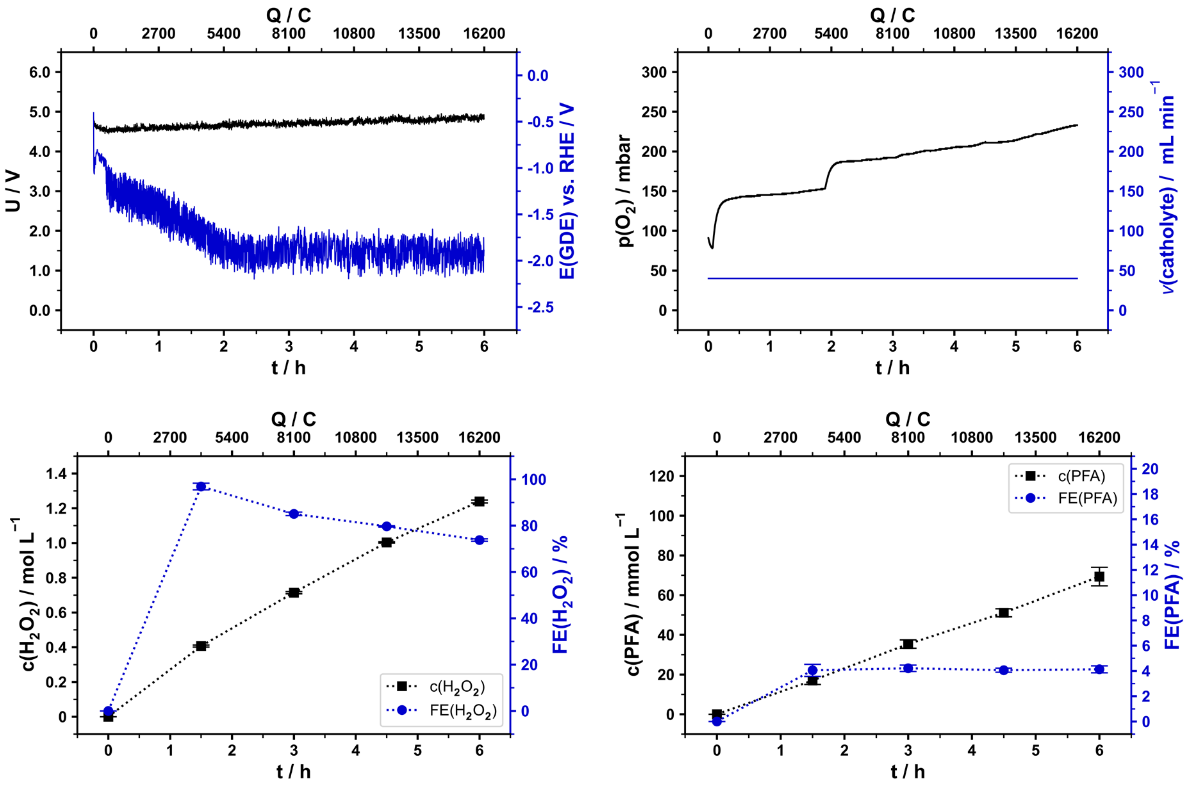

Figure S17: Data for electrolysis (R1), experimental details are provided in section 1.5 and results in Table S14 and Table S15.

Table S26: Concentration and Faradaic efficiency (FE) of H_2O_2 and PFA in the catholyte for electrolysis (R2) with 0.2 mol L^{-1} KH_2PO_4 / K_2HPO_4 containing 0.5 mol L^{-1} HCOOK / HCOOH in 1 wt% H_3PO_4 as starting electrolyte (cf. Figure S18).

t / h	$c(H_2O_2)$ / mol L^{-1}	$FE(H_2O_2)$ / %	c(PFA) / mmol L^{-1}	FE(PFA) / %
1.5	0.3763 ± 0.0013	89.6 ± 0.3	16.7 ± 1.1	4.0 ± 0.3
3	0.6733 ± 0.0015	80.2 ± 0.2	34.3 ± 2.1	4.1 ± 0.3
4.5	0.8867 ± 0.0034	70.4 ± 0.3	52.0 ± 2.4	4.1 ± 0.2
6	1.0311 ± 0.0070	61.4 ± 0.5	64.4 ± 3.9	3.8 ± 0.3

Table S27: pH for electrolysis (R2) with 0.2 mol L^{-1} KH_2PO_4 / K_2HPO_4 containing 0.5 mol L^{-1} HCOOK / HCOOH in 1 wt% H_3PO_4 as starting electrolyte.

t / h	pH (catholyte)	pH (anolyte)
0	4.05	0.33
1.5	4.01	0.30
3	3.97	0.29
4.5	3.96	0.28
6	3.96	0.27

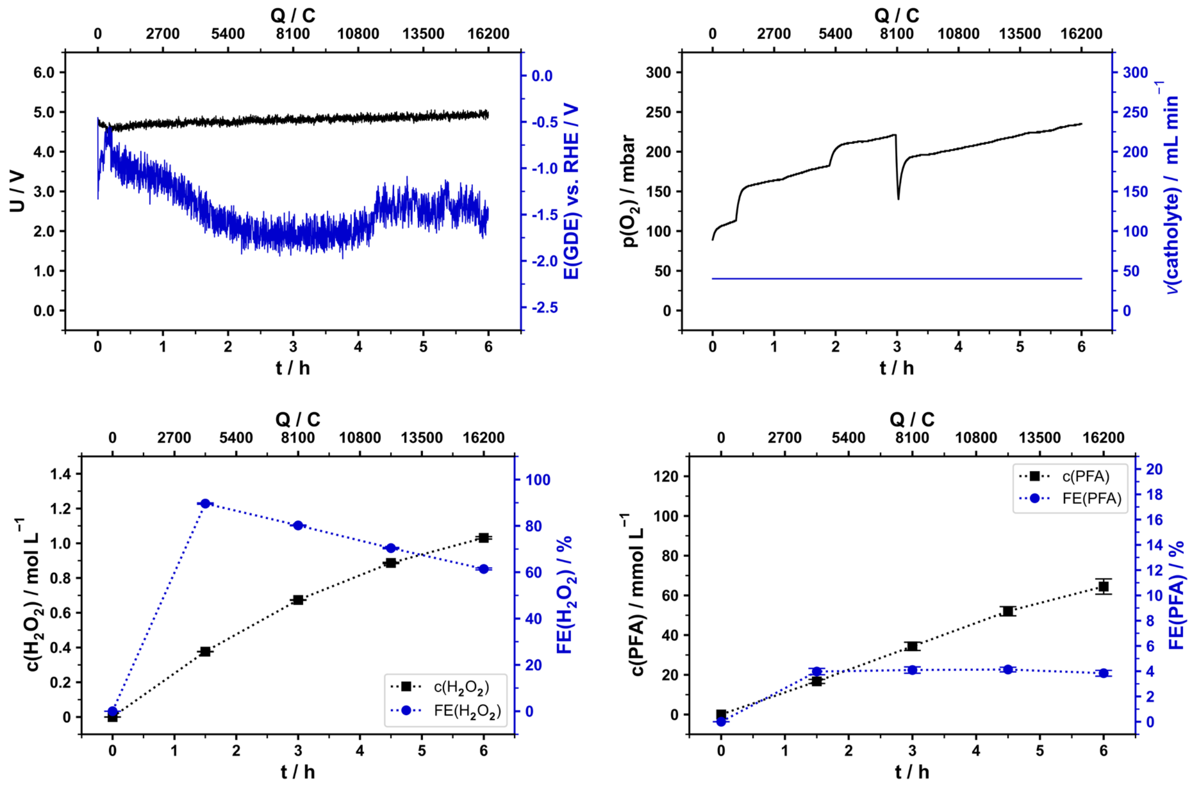

Figure S18: Data for electrolysis (R2), experimental details are provided in section 1.5 and results in Table S14 and Table S15.

Table S28: Concentration and Faradaic efficiency (FE) of H_2O_2 and PFA in the catholyte for electrolysis (R3) with 0.2 mol L^{-1} KH_2PO_4 / K_2HPO_4 containing 0.5 mol L^{-1} HCOOK / HCOOH in 1 wt% H_3PO_4 as starting electrolyte (cf. Figure S19).

t / h	$c(H_2O_2)$ / mol L^{-1}	$FE(H_2O_2)$ / %	c(PFA) / mmol L^{-1}	FE(PFA) / %
1.5	0.3654 ± 0.0008	87.1 ± 0.2	15.5 ± 0.5	3.7 ± 0.2
3	0.6767 ± 0.0011	80.6 ± 1.3	39.0 ± 3.0	4.6 ± 0.4
4.5	0.9578 ± 0.0039	76.1 ± 0.4	59.6 ± 1.6	4.7 ± 0.2
6	1.1722 ± 0.0051	69.8 ± 0.4	72.9 ± 0.8	4.3 ± 0.1

Table S29: pH for electrolysis (R3) with 0.2 mol L^{-1} KH_2PO_4 / K_2HPO_4 containing 0.5 mol L^{-1} HCOOK / HCOOH in 1 wt% H_3PO_4 as starting electrolyte.

t / h	pH (catholyte)	pH (anolyte)
0	4.08	0.34
1.5	4.03	0.33
3	3.99	0.30
4.5	3.99	0.29
6	3.98	0.27

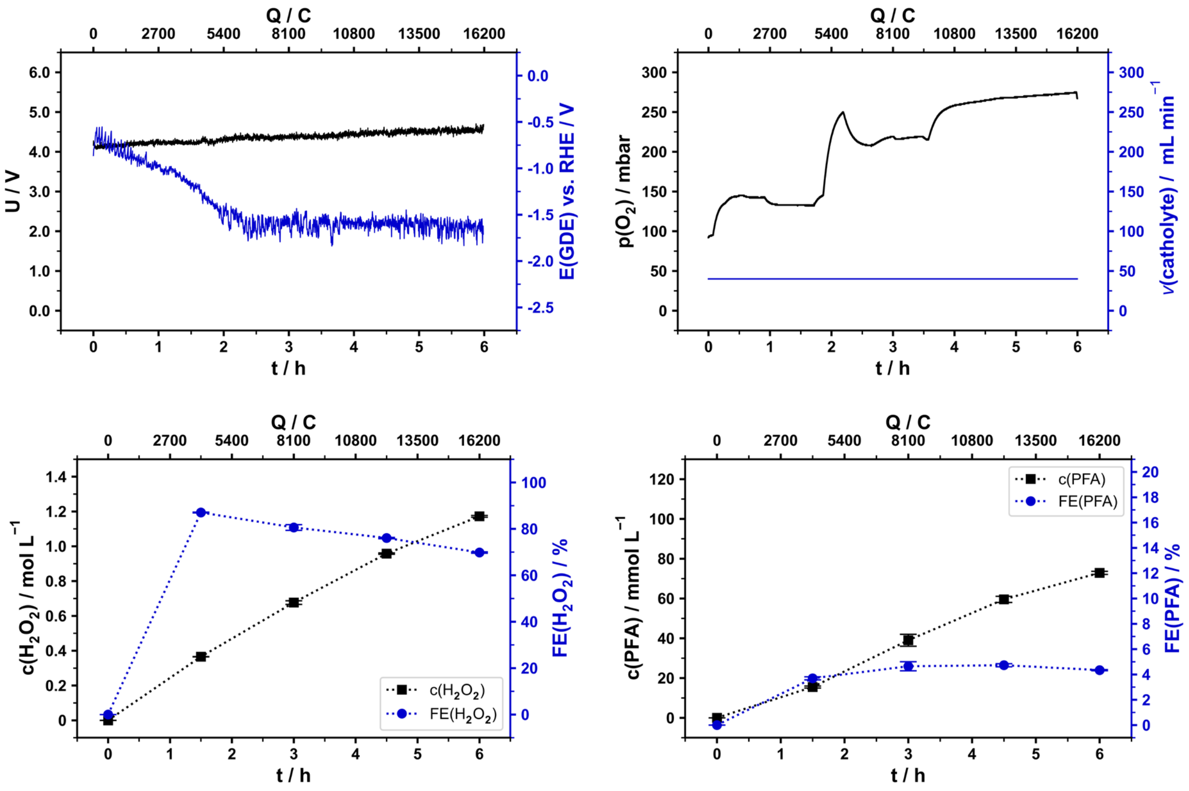

Figure S19: Data for electrolysis (R3), experimental details are provided in section 1.5 and results Table S14 and Table S15.

Table S30: Concentration and Faradaic efficiency (FE) of H_2O_2 and PFA in the catholyte for electrolysis (R4) with 0.2 mol L^{-1} KH_2PO_4 / K_2HPO_4 containing 0.5 mol L^{-1} HCOOK / HCOOH in 1 wt% H_3PO_4 as starting electrolyte (cf. Figure S18).

t / h	$c(H_2O_2)$ / mol L^{-1}	$FE(H_2O_2)$ / %	c(PFA) / mmol L^{-1}	FE(PFA) / %
1.5	0.3600 ± 0.0013	85.8 ± 0.3	17.7 ± 0.8	4.2 ± 0.2
3	0.6583 ± 0.0039	78.4 ± 0.5	36.3 ± 1.2	4.3 ± 0.2
4.5	0.9044 ± 0.0039	71.8 ± 0.4	59.6 ± 2.1	4.7 ± 0.2
6	1.0833 ± 0.0034	64.5 ± 0.2	67.6 ± 0.8	4.0 ± 0.1

Table S31: pH for electrolysis (R4) with 0.2 mol L^{-1} KH_2PO_4 / K_2HPO_4 containing 0.5 mol L^{-1} HCOOK / HCOOH in 1 wt% H_3PO_4 as starting electrolyte.

t / h	pH (catholyte)	pH (anolyte)
0	4.07	0.35
1.5	4.02	0.33
3	3.98	0.31
4.5	3.99	0.31
6	4.00	0.27

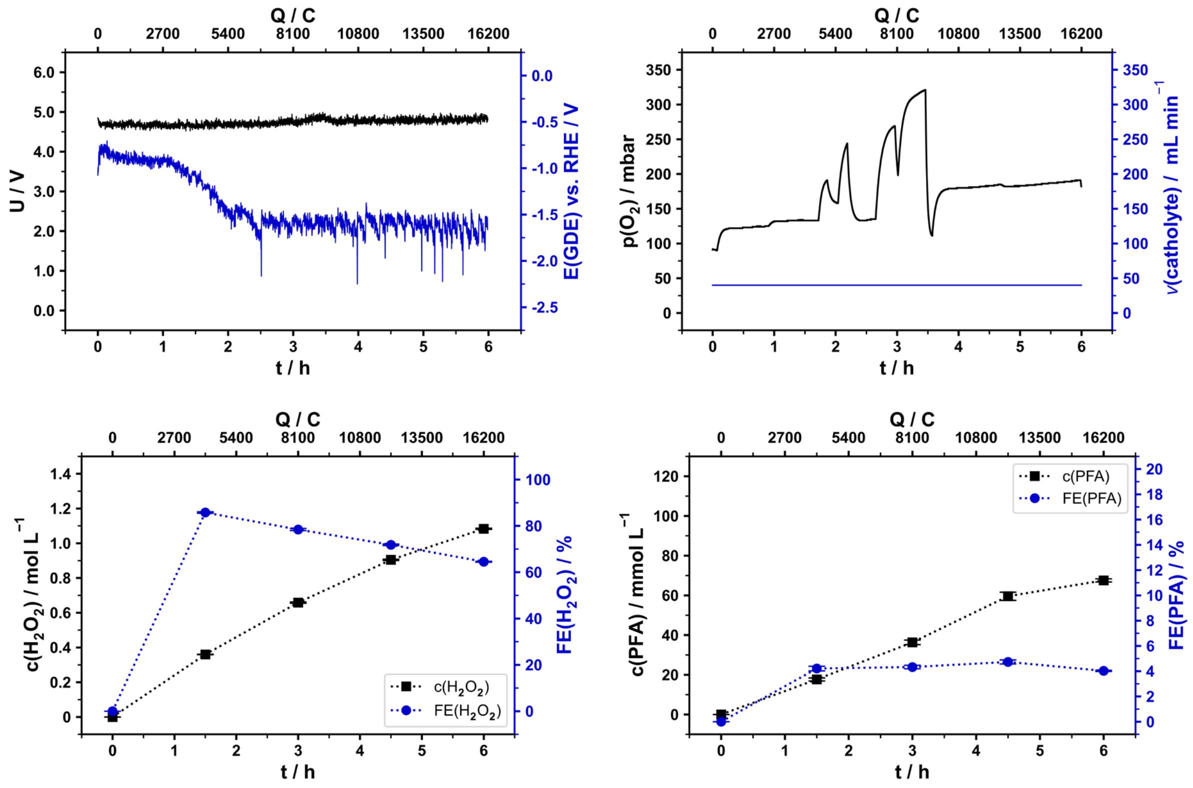

Figure S20: Data for electrolysis (R4), experimental details are provided in section 1.5 and results in Table S14 and Table S15.

3.3 Pictures of GDE before and after electrolysis

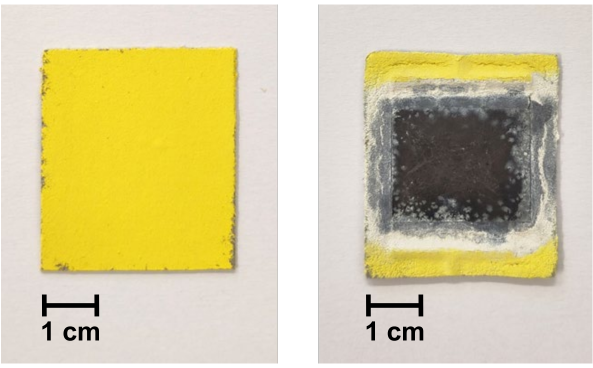

Figure S21: Exemplary pictures of a self-fabricated Bi_2O_3 based GDE before (left) and after (right) electrolysis. Details of the fabrication process are provided in section 1.2.1, details on the electrolysis conditions in section 1.4.

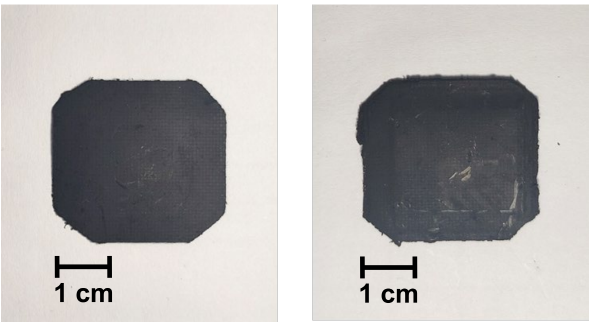

Figure S22: Exemplary pictures of a self-fabricated Carbon based GDE before (left) and after (right) electrolysis. Details of the fabrication process are provided in section 1.2.2, details on the electrolysis conditions in section 1.5.

3.4 SEM images of GDE before and after electrolysis

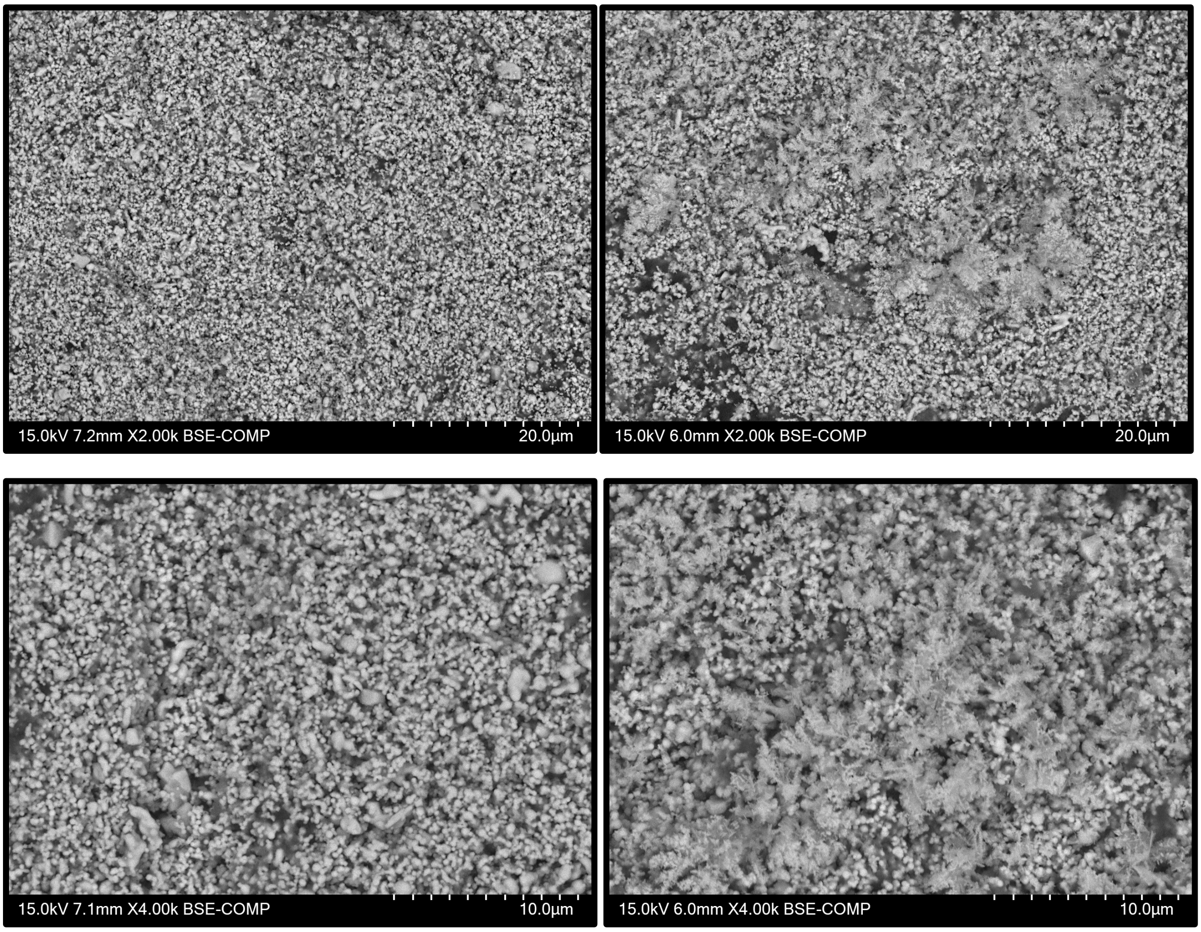

Figure S23: Exemplary SEM images (BSE, bright particles are Bi, section 1.8) of the GDE for formate electrosynthesis before (left) and after (right) electrolysis.

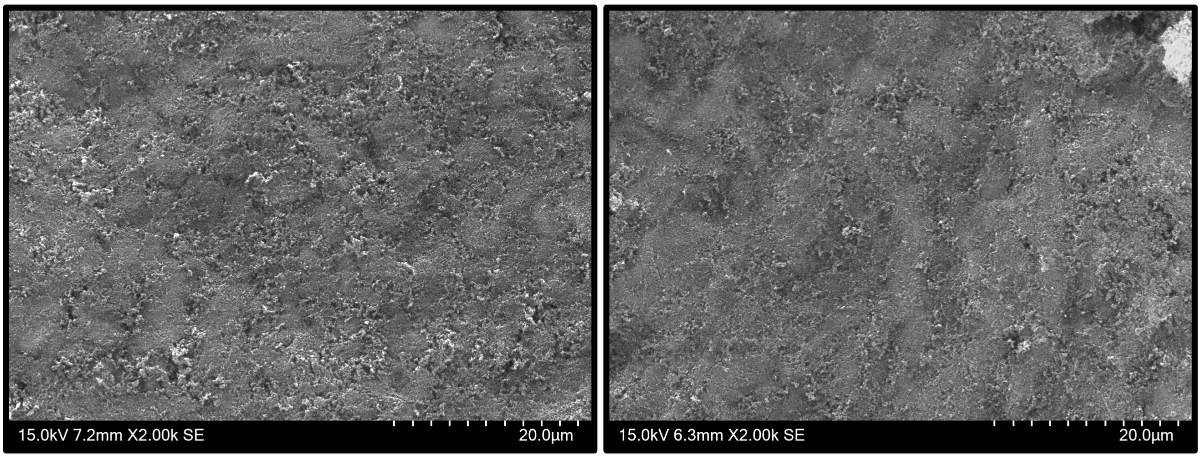

Figure S24: Exemplary SEM image (SE, cf. section 1.8) of the GDE for H_2O_2 / PFA (electro)synthesis before (left) and after (right) electrolysis.

3.5 Contact angle of GDE before and after electrolysis

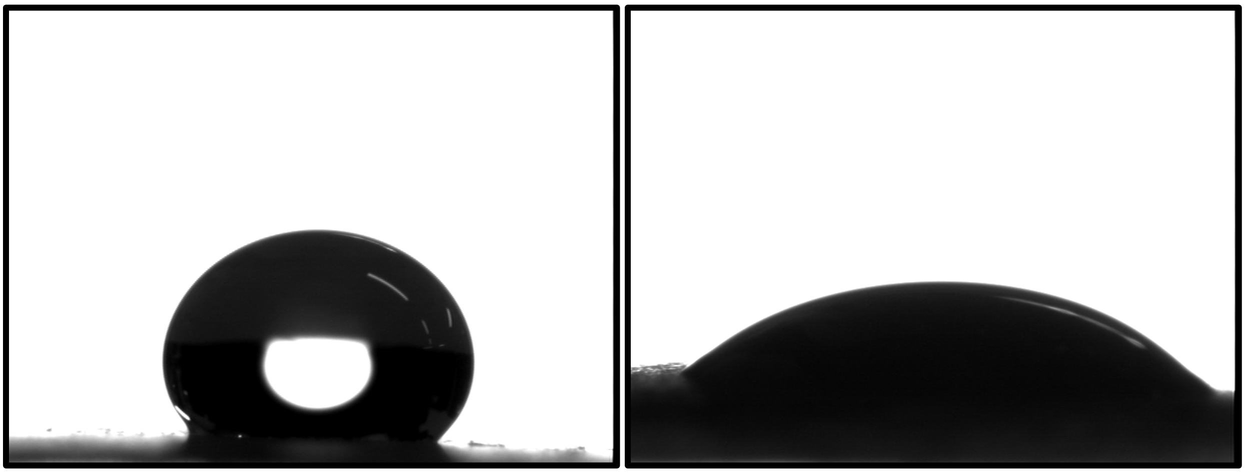

Figure S25: Exemplary images for contact angle θ determination (cf. section 1.9) of the Bi_2O_3 based GDE for formate electrosynthesis before (left) and after (right) electrolysis (cf. Table S32).

Table S32: Exemplary contact angles θ of a Bi_2O_3 based GDE before and after application for formate electrosynthesis**.**

	θ (left) / °	θ (right) / °	θ (average) / °
Before electrolysis	136.34	136.02	136.2 ± 0.3
After electrolysis	46.26	48.71	47.5 ± 1.8

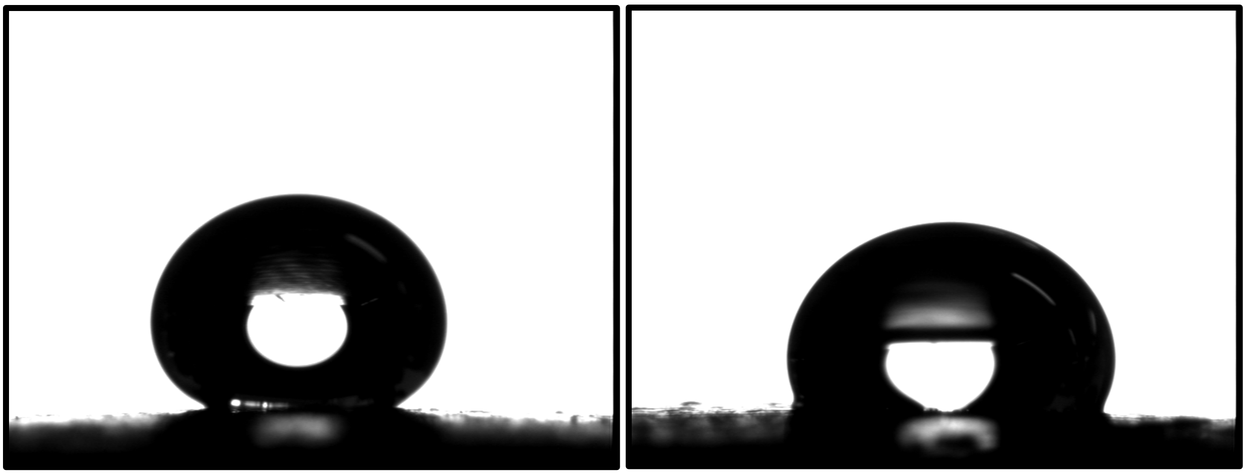

Figure S26: Exemplary images for contact angle θ determination (cf. section 1.9) of the carbon based GDE for H_2O_2 / PFA (electro)synthesis before (left) and after (right) electrolysis (cf. Table S33).

Table S33: Exemplary contact angles θ of a carbon based GDE before and after application for H_2O_2 / PFA (electro)synthesis**.**

	θ (left) / °	θ (right) / °	θ (average) / °
Before electrolysis	142.77	142.66	142.72 ± 0.08
After electrolysis	116.24	116.52	116.4 ± 0.2

3.6 X-ray diffraction of GDE before and after electrolysis

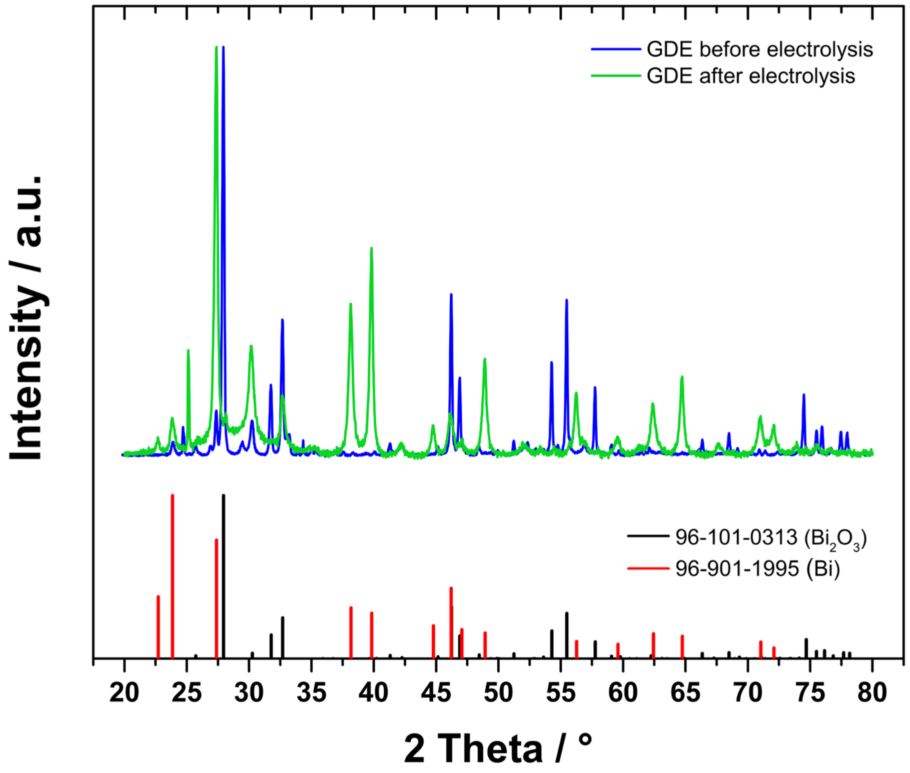

Figure S27: XRD data of the Bi_2O_3 based GDE for formate electrosynthesis before and after electrolysis as well as peak positions of Bi_2O_3 (96-101-0313) and Bi (96-901-1995) from the PDF-2 database (International Centre of Diffraction Data - ICDD) for comparison.
